# Tough Hydrogels for Load‐Bearing Applications

**DOI:** 10.1002/advs.202307404

**Published:** 2024-01-15

**Authors:** Nika Petelinšek, Stefan Mommer

**Affiliations:** ^1^ Macromolecular Engineering Laboratory Department of Mechanical and Process Engineering ETH Zurich Sonneggstrasse 3 Zurich 8092 Switzerland

**Keywords:** elastic modulus, energy dissipation, fracture energy, load‐bearing applications, tough hydrogels

## Abstract

Tough hydrogels have emerged as a promising class of materials to target load‐bearing applications, where the material has to resist multiple cycles of extreme mechanical impact. A variety of chemical interactions and network architectures are used to enhance the mechanical properties and fracture mechanics of hydrogels making them stiffer and tougher. In recent years, the mechanical properties of tough, high‐performance hydrogels have been benchmarked, however, this is often incomplete as important variables like water content are largely ignored. In this review, the aim is to clarify the reported mechanical properties of state‐of‐the‐art tough hydrogels by providing a comprehensive library of fracture and mechanical property data. First, common methods for mechanical characterization of such high‐performance hydrogels are introduced. Then, various modes of energy dissipation to obtain tough hydrogels are discussed and used to categorize the individual datasets helping to asses the material's (fracture) mechanical properties. Finally, current applications are considered, tough high‐performance hydrogels are compared with existing materials, and promising future opportunities are discussed.

## Graphical abstract

In this review, a comprehensive dataset on tough hydrogels with extreme (fracture) mechanical properties is presented, which have combined a wide variety of energy dissipation modes. The gathered data is decoded according to the water content of the individual samples. Outstanding hydrogel examples are further discussed, trends, as well as applications, and compare current cutting‐edge materials with commercial ones.

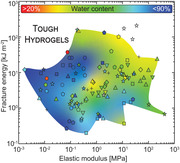

## 1 Motivation

Hydrogels are cross‐linked, water‐based polymer networks with reversible swelling, tunable porosity, elasticity, toughness, stiffness, and flexibility. The global market for hydrogels was valued 25.2 billion U.S. dollars in 2021 and is projected to reach 45.2 billion U.S. dollars by 2030 with major product segments in personal care and hygiene (sanitary pads, diapers), pharmaceuticals and healthcare, contact lenses, and agriculture.^[^ ^1^ ^]^ In academic research, hydrogels are commonly applied in tissue engineering and regenerative medicine,^[^ ^2^, ^3^, ^4^ ^]^ additive manufacturing,^[^ ^5^, ^6^ ^]^ drug delivery,^[^ ^7^ ^]^ as (bio)sensors,^[^ ^8^, ^9^ ^]^ underwater adhesives,^[^ ^10^ ^]^ actuators, or soft robots,^[^ ^11^, ^12^ ^]^ in information storage,^[^ ^13^ ^]^ or photonics.^[^ ^14^ ^]^ Each application area requires a careful adjustment of the microstructural features of the hydrogel in order to engineer the material with suitable macroscopic mechanical properties. This connection between the physicochemical properties on a molecular level and the mechanical properties of the macroscopic object is commonly known as the structure–property relationship. Understanding the structure–property relationship of a material is of critical importance to predict material behavior and, therefore, has enabled researchers to engineer innovative hydrogel materials.

**Figure 1:**
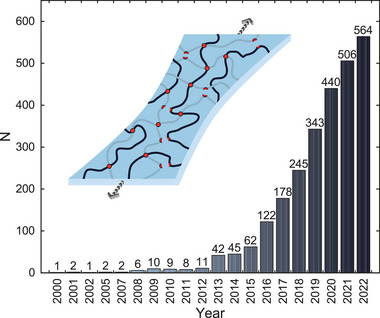
Number of publications N that meet the search criteria of “tough hydrogels” within the last 20 years. Citation Report graphic is derived from Clarivate Web of Science, Copyright Clarivate 2023. All rights reserved.

Recently, there has been a growing interest to mimic highly stiff natural tissues, such as tendons or cartilage, whose elastic moduli lie in the MPa and GPa range, respectively.^[^ ^15^, ^16^ ^]^ As a result, the field of hydrogels has experienced a shift toward tough hydrogels with enhanced mechanical properties, such as high stiffness, (fracture) toughness, and elasticity, which boost the hydrogel's resilience toward extreme mechanical impacts and load‐bearing applications. To fabricate such tough hydrogels, strategies to dissipate energy throughout the network are necessary. Efficient energy dissipation has been achieved by using sacrificial bonds that transform the kinetic energy of a mechanical impact into chemical energy to (reversibly) break the sacrificial bonds. This strategy of energy dissipation has enabled a rich variety of tough hydrogels with a steadily growing interest in these materials being reflected in the amount of articles published throughout the past 20 years (**Figure** **1**).

The topic gained traction from 2003 onward, when the groups of Gong, Creton, Schmidt, and others elaborated on the concepts of double networks and nanocomposite hydrogels.^[^ ^17^, ^18^, ^19^, ^20^, ^21^, ^22^ ^]^ Specifically, Gong et al. demonstrated for the first time how hydrogels with extremely high mechanical strength can be accessed by using a double network architecture.^[^ ^17^ ^]^ In 2012, Suo and co‐workers presented double networks combining covalent and physical cross‐links,^[^ ^23^ ^]^ which —together with the previous works— pioneered this relatively young area of research, sparking a surge of studies thereafter. In the following year, the number of publications quadrupled and just in 2022 reached 564 publications.

Since it is not quantity, but quality that counts, many reports have benchmarked their results in Ashby plots as a means of data evaluation. In said plots, a combination of two chosen material properties are plotted to identify the best parameter combination for a specific application. While useful in general, such plots often entertain selective data choices and omit important, tertiary properties or parameters. For tough hydrogels in particular, such an important parameter is the water content, as high water contents weaken mechanical properties, while low ones can enhance them. Consequently, benchmarks may fall short of their mandate to deliver a fair comparison, giving an arguably distorted view on the matter.

In this review, we set out to present a detailed and comprehensive comparison between individual tough hydrogel systems. Importantly, we decode the plotted results according to the water content of the respective hydrogels, which enables us to discuss and showcase outstanding contributions to the field. We limit ourselves to materials that have extensively been characterized regarding their stiffness, elasticity and fracture toughness. The fracture toughness is a particular important factor as it highlights the hydrogel's capacity to resist crack propagation during load‐bearing applications.

First, we introduce mechanical characterizations of tough hydrogels to establish a fundamental understanding of their mechanical properties. Next, various modes of energy dissipation as a key factor to access tough hydrogels are examined. Many tough hydrogels have emerged through a clever combination of various dissipation modes, thus, an overview will facilitate the classification of tough hydrogels afterward. The central part of this review deals with mechanical property data of tough hydrogels, where datasets are summarized and presented to elucidate correlations and trends. Further, current applications of tough hydrogels and comparative values in naturally occurring materials are discussed to underline individual examples of outstanding importance. Finally, we briefly reflect upon the current distribution of mechanical properties and compare with existing materials with an eye on further potential applications that are currently being less explored. In the closing remarks, we summarize the individual design strategies for tough hydrogels, discuss some of the advantages, limitations and applicabilities and end the review with a more general view on the field and recommendations for the future.

## 2 Fracture Mechanical Characterization

Analogous to other materials, analyzing the mechanical properties of tough hydrogels is essential to assess their capacity for specific engineering applications. Generally speaking, the mechanical properties describe the hydrogel's behavior when subjected to load and determine its ability to withstand, deform, and return to its original shape as a direct consequence of such loads. The precise characterization and assessment of the material's mechanical properties further allows prediction of its performance over time and under various environmental conditions. While the mechanical loading of hydrogels can occur in various modes of deformation (shearing, tensile, compression, bend, or hardness testing), we will primarily focus on tensile tests.

**Figure 2:**
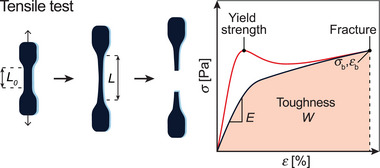
Uniaxial tensile test experiment of a plastically deforming material (red) and an elastic hydrogel (black).

Tensile tests have been extensively used for the characterization of tough hydrogels and are commonly carried out on a universal testing machine. In a typical tensile test experiment, a dumbbell‐shaped hydrogel specimen (sometimes referred to as dogbone) is clamped with a fixed distance *L*_0_ also known as gauge length (**Figure** **2**). Next, the tensile force is recorded while one clamp is displaced with a constant velocity resulting in an elongation *L* of the deforming sample. The tensile strain ε can be calculated either from the stretch ratio λ or directly through *L* and *L*_0_ according to

(1) $$\varepsilon =\lambda -1=\frac{L}{L_{0}}-1=\frac{L-L_{0}}{L_{0}}=\frac{\Delta L}{L_{0}}$$

While the tensile strain is expressed in %, the stretch ratio λ is a dimensionless number. The nominal stress σ that is applied to the sample, correlates with the applied force *F* and the sample's original cross‐sectional area *A*_0_ through

(2) $$\sigma =\frac{F}{A_{0}}$$

and is therefore expressed in units of N m^−2^ or Pascals (Pa). Note that this is different to the true stress, which relates the applied force *F* to the actual cross‐sectional area *A* during the deformation process of the sample. Most publications, however, have focused on the nominal stress only, since the true stress is predominatly used for materials with extensive plastic deformation.

The overall purpose of the tensile test is to evaluate the ductility and strength of the material. From the data collected during the test, various parameters are obtained, such as the elastic modulus, the ultimate tensile strength, yield strength, elongation at break, or the toughness. The elastic modulus *E*, also known as Young's modulus, reflects the hydrogel's ability to resist deformation and is therefore a measure of stiffness. It is defined as the ratio of the tensile stress to the resulting strain in the elastic region of the material. Hence, it is calculated as the slope of the linear portion of the stress–strain curve generally spanning the strain range from 0–10%, in more rare cases a maximum of 15% strain is considered. The yield strength is the amount of stress that a material can undergo before transitioning from reversible elastic to irreversible plastic deformation, which is also known as yielding. After yielding, conventional polymer samples often undergo strain‐softening, caused by the molecular chains being drawn along the tensile strain axis. Once the polymer chains are oriented, the material may experience strain‐hardening due to the stress being fully concentrated onto the aligned polymer chains, followed by fracture. In hydrogels and more generally soft elastic networks, the various cross‐links may prevent chains from being aligned, which is why they often do not plastically yield and do not exhibit a true yield strength. Consequently, the fracture stress at break is deemed more important and is measured where ultimate tensile failure occurs (σ_*b*_).

Finally, the toughness *W* measures a material's ability to absorb energy before breaking and therefore equals to the work of extension until failure. Sometimes referred to as strain‐energy density, the toughness *W* can be obtained through integration of the area under the stress‐strain (or stress‐stretch) curve using the equation below (Figure 2):

(3) $$W=\int_{\varepsilon }^{\varepsilon_{max}}\sigma \left(\varepsilon \right)d\varepsilon \;\mathrm{or}\;W=\int_{\lambda }^{\lambda_{max}}\sigma \left(\lambda \right)d\lambda$$

Note that this is different than the inner elastic energy *U*, which is calculated from the area under the force–extension curve instead. Toughness is expressed in MJ m^−3^ and represents an important property for materials that are subjected to high impact loading or other types of dynamic loading.

**Figure 3:**
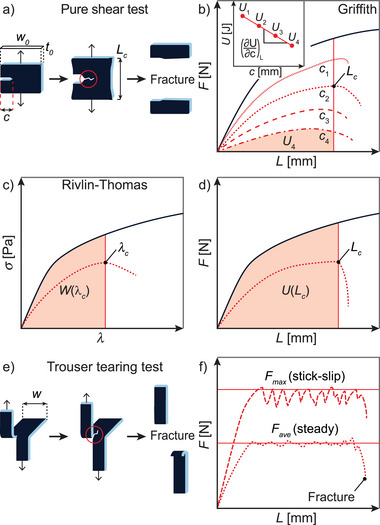
a) Pure shear test and corresponding force–displacement plots according to b) Griffith and c+d) Rivlin‐Thomas using unnotched samples (black lines) and single notched specimen with crack lengths *c* (dashed lines). e) Trouser tearing test and f) corresponding force–displacement plot showing stick‐slip (dashed lines) and steady tearing (dotted lines).

In the real world, many materials may contain or develop small cracks and defects that arise from the manufacturing process or through aging. Since many applications often require the designed material to undergo a multitude of high impact loading cycles, it is crucial that such cracks do not propagate to prevent catastrophic failure. As a consequence, the determination of fracture toughness has become increasingly important for the characterization of tough hydrogels. Unlike the toughness *W* (material's resistance to fracture), fracture toughness specifically measures a material's resistance to crack propagation. By measuring fracture toughness, the performance of a material in the presence of small cracks can be evaluated and whether it can withstand load‐bearing conditions. Fracture toughness is measured in various modes, most commonly with a pure shear test or a trouser tearing test (**Figure** **3**).

In the pure shear test, a single edge notched sample specimen with a crack of length *c* is pulled in one direction until the crack starts propagating (Figure 3a). Following the Griffith criterion, a sample whose crack propagates from length *c* to *c* + *dc* suffers a loss of stored elastic energy *U*.^[^ ^24^ ^]^ This difference in elastic energy is transformed into free surface energy required to create new crack surface; it can be described by the energy release rate *G* according to

(4) $$G=-\frac{1}{t_{0}}{\left(\frac{\partial U}{\partial c}\right)}_{L}$$

with *t*_0_, the thickness of the test specimen and *G* being expressed in J m^−2^. Since crack propagation is hard to control and often results in sudden catastrophic failure of the sample, multiple samples with varying crack lengths *c* are measured consecutively (Figure 3b). In such a scenario, each sample exhibits a different elastic energy *U*, which is quantified by integrating the area under the curve until a selected displacement length *L*. Next, the calculated elastic energies *U*_1_ to *U*_4_ (unit Joule J) are plotted against the crack length *c* to deliver the energy release rate *G* for the sample to be extended to this particular *L* value (Figure 3b, inset). Finally, when the displacement length *L* is chosen as such that at least one sample fractures (*L* = *L*_*c*_), the energy release rate becomes equal to the critical energy release rate *G*_*c*_ also known as fracture energy or fracture toughness Γ.

(5) $$G\left(L_{c}\right)=G_{c}=\Gamma$$

Beside the approach discussed above, Rivlin and Thomas introduced a method to determine the fracture energy by measuring an unnotched and a single notched sample.^[^ ^25^, ^26^ ^]^ During the measurement, the stress of a notched sample is recorded with progressing stretch ratio until crack propagation occurs marking the critical stretch ratio λ_*c*_ (Figure 3c). Next, the work of extension of the unnotched sample is calculated up to λ_*c*_ so that the fracture energy can be calculated following

(6) $$\Gamma =W\left(\lambda_{c}\right)h_{0}$$

with *h*_0_, the initial length of the sample (identical to *L*_0_). Alternatively, notched and unnotched specimen can be measured and compared in a force–displacement plot (Figure 3d). Again, a force is applied to displace a notched sample until the onset of crack propagation is observed at the critical displacement length *L*_*c*_. Integrating the area under the extension curve of the unnotched sample up to *L*_*c*_ gives rise to the critical elastic energy *U*(*L*_*c*_). As a result, Equation (6) can be rewritten to

(7) $$\Gamma =\frac{U(L_{c})}{A_{0}}$$

with *A*_0_ = *w*_0_ *t*_0_ being the cross‐sectional area, width, and thickness of the pristine unstretched sample, respectively.^[^ ^23^ ^]^

Another test to investigate fracture properties of (soft) materials is the trouser tearing test (Figure 3e).^[^ ^25^, ^27^ ^]^ A rectangular sample specimen of width *w* is cut along its length to give two identical legs, which are clamped and pulled in opposite direction with a force *F*.^[^ ^28^ ^]^ At a certain critical displacement length the crack will start to propagate and the subsequent tearing process can be catergorized into steady and stick‐slip tearing (Figure 3f).^[^ ^29^ ^]^ For steady‐state tearing, fluctuations of the tearing force remain low and the tearing behavior is best represented by the average tearing force *F*_*ave*_. For the stick‐slip tearing mode, distinct fluctuations of the tearing force occur, the latter regularly reaching a maximum followed by a sudden and rapid decline to a local minimum. In this case, the tearing energy is often derived from the maximum tearing force *F*_*max*_. In both cases, the tearing energy *T* (kJ m^−2^), which is synonymous with the fracture energy Γ, can be calculated through Equation (8):

(8) $$\Gamma =T=\frac{2F_{ave/max}}{t_{0}}$$

Both the pure shear test as well as the trouser tearing test (using Equations (6)–(8)) share the unique feature that if the right geometric constraints for the sample are met, the calculated energies are essentially independent from the crack length *c*. As a result, these type of fracture tests are convenient to study fracture mechanics of soft materials and have thus become very popular within the soft matter community.

**Figure 4:**
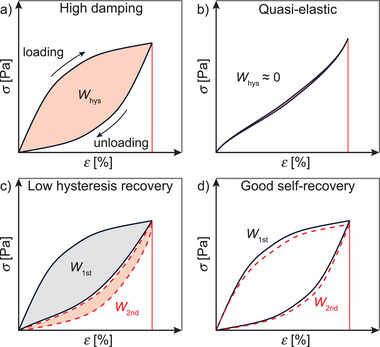
Cyclic tensile tests of tough hydrogels showing stress–strain diagrams of a sample with a) high damping capacity, b) quasi‐elastic behavior, c) low hysteresis recovery, and d) good self‐recovery.

Additionally and often due to their non‐covalent nature, tough hydrogels exhibit pronounced self‐healing and self‐recovering properties. To characterize those, cyclic tensile tests are carried out for which the sample undergoes successive loading and unloading phases. The maximum strain of the tensile cycle is chosen to be lower than the failure strain of the sample. After the loading phase, the stress is released and the hydrogel ideally contracts to its original shape (**Figure** **4**a,b). Such a cyclic tensile test allows the calculation of the hysteresis work *W*_*hys*_, which describes the energy that is lost or dissipated during one deformation cycle (loading & unloading). Whether this work is desirably large or small depends on the hydrogel's intented use. For instance, in damping applications a system is required to absorb and dissipate energy to be able to mitigate a shock or vibration and enhance system stability. In this case, larger values for *W*_*hys*_ are needed (Figure 4a). Conversely, sometimes lower dissipation energies are clearly preferred, for example, when a minimization of energy loss is critical to provide resistance to multiple loading cycles (Figure 4b).

In general, many tensile cycles are performed successively to assess the performance decrease with progressing number of cycles, which is also known as fatigue resistance. The immediate second tensile cycle often returns only a fraction of the energy dissipated in the first one, with their arithmetic ratio expressing the hysteresis recovery (Figure 4c–d):

(9) $$h_{r}=\frac{W_{2nd}}{W_{1st}}$$

## 3 Modes of Energy Dissipation

**Figure 5:**
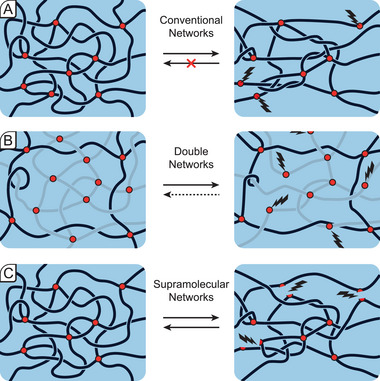
Mechanical stress causing energy dissipation in a) conventional hydrogels, b) double networks, and c) non‐covalently cross‐linked networks.

Conventional hydrogels often suffer from weak mechanical properties and display brittle and unstable behavior limiting their scope for load‐bearing applications. Such networks consist of functionalized polymers with covalent cross‐links at fixed positions along the polymer backbone, their energy being on the order of 80 kcal mol^−1^ for a standard carbon–carbon bond (**Figure** **5**A).^[^ ^30^ ^]^ Upon deformation, tensile stress is concentrated on the closest neighbouring cross‐links, eventually leading to their rupture and material failure. Increasing the toughness of conventional hydrogels is often attempted by making longer network strands, which however reduces stiffness and fracture strength of the gels. To fabricate hydrogels that are stiff and tough at the same time, mechanisms to dissipate energy are essential, since they allow the gel to reversibly recover from a mechanical impact. Hence, designing high‐performance hydrogels requires molecular strategies to enable efficient energy dissipation throughout the hydrogel network.

Energy dissipation is often achieved through the introduction of sacrificial bonds, which occur in two categories: i) a second more brittle network is polymerized within a first loosely cross‐linked network (double networks)^[^ ^17^ ^]^ or ii) cross‐links are made from supramolecular transient interactions, which can be reversibly broken and reformed (Figure 5b,c). In the first case, the material is stretched and a share of the bonds in the second, more brittle network are broken leading to an increased dissipation of the energy from mechanical impact. In the second case, the sacrificial bonds are of non‐covalent nature and can reversibly break and reform. Non‐covalent interactions include, but are not limited to, metal–ligand, ionic interactions, hydrogen bonding, microphase separation and hydrophobic interactions, polymer–nanomaterial adsorption, host–guest complexation, or supramolecular self‐assembly. As we will later see, cutting‐edge materials often combine both sacrificial bond categories, as well as multiple non‐covalent interactions to maximize the energy dissipation in the network. However, first we briefly discuss the most frequent modes of energy dissipation at hand.

**Figure 6:**
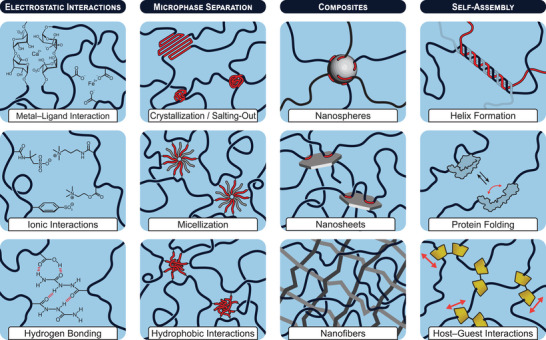
An overview of energy dissipation modes each represented by a specific molecular interactions/architectures, which have served the fabrication of tough hydrogels. Individual modes of energy dissipation are categorized according to eletrostatic interactions, microphase saparation, composites, or self‐assembly.

Metal–ligand interactions arise when a ligand donates a lone electron pair to empty orbitals of a metal ion, forming a coordinate bond that is dynamic as opposed to a covalent bonds (**Figure** **6**).^[^ ^31^ ^]^ Typical ligands are small organic molecules that contain at least one free pair of electrons and therefore act as Lewis bases. Such coordinate bonds can be formed by a variety of metal ions and neutral or anionic ligands covering a broad range of binding constants (*K*_*a*_ = 10^3^–10^40^) characteristic of strong covalent down to weaker non‐covalent interactions.^[^ ^32^ ^]^ The complex stability generally increases with higher oxidation state and smaller size of central metal cation, for instance, following the Irvin‐Williams series: Mn^2 +^ < Fe^2 +^ < Co^2 +^ < Ni^2 +^ < Cu^2 +^ > Zn^2 +^, where copper ions form complexes with the highest equilibrium constants.^[^ ^33^ ^]^ On the ligand side, complex stability increases with i) the ligand's basicity facilitating the donation of lone electron pairs and ii) its denticity describing the number of ligand binding sites involved in the metal coordination (chelation).^[^ ^31^ ^]^ For example, a monodentate imidazole ligand forms metal–ligand complexes with substantially lower stability constants than its bidentate equivalent, histidine.^[^ ^34^ ^]^ Tough polymer networks based on metal–ligand coordination are typically formed by ligand‐containing polymers that undergo chelation once a suitable metal cation is introduced. Beside the use of natural metal‐chelating polysaccharides, such as alginate^[^ ^23^, ^35^, ^36^, ^37^, ^38^, ^39^, ^40^, ^41^, ^42^ ^]^ or carrageenan,^[^ ^43^, ^44^, ^45^, ^46^ ^]^ prominent ligands include carboxylates,^[^ ^47^, ^48^, ^49^ ^]^ imidazole,^[^ ^50^, ^51^ ^]^ among others.^[^ ^31^, ^52^ ^]^ Consequently, metal–ligand interactions have successfully produced a variety of tough hydrogels with high capacity for energy dissipation.

Ionic interactions arise from electrostatic attraction between two oppositely charged species (Figure 6). The strength of an ionic bond in an aqueous environment ranges between 5–10 kcal mol^−1^ and scales directly with the charges of the interacting species, while being inversely proportional to the distance between them.^[^ ^30^ ^]^ Ionically cross‐linked hydrogels can be obtained through the interactions between cationic and anionic functional groups of polymers. This process is commonly referred to as polyelectrolyte complex formation.^[^ ^53^ ^]^ Alternatively, ionic functionalities of polymers can be cross‐linked with small molecules that carry multiple opposite charges.^[^ ^54^ ^]^ For example, the natural polysaccharide chitosan (CS) carries positively charged ammonium groups (pK_*a*_ ≈ 6.5), which can interact with negatively charged polymers or organic polyanions such as sodium triphosphate or sodium phytate to form cross‐linked networks.^[^ ^54^, ^55^, ^56^ ^]^ The hydrogel properties may then be adjusted through polymer concentration, degree of ionization of the charged groups, pH, ionic strength, and temperature.^[^ ^54^ ^]^

Many tough hydrogels based on ionic interactions, however, have employed polyampholytes, which are polymers furnished with both cationic and anionic groups.^[^ ^57^ ^]^ Representative cationic monomers are 3‐(methacryloylamino)propyl‐trimethylammonium chloride (MPTC) or dimethylaminoethyl acrylate hydrochloride (DMAEA‐Q), while popular anionic monomers include sodium acrylate or sodium *p*‐styrenesulphonate (NaSS).^[^ ^58^, ^59^, ^60^, ^61^, ^62^ ^]^ The random distribution of ionic groups within the polymer backbone leads to ionic interactions of various strengths, where stronger ones may act as persistent cross‐links, while weaker ones are more dynamic serving as sacrificial bonds within the network.^[^ ^58^ ^]^ Consequently, ionic interactions have been used in various ways to mediate reversible cross‐linking for the fabrication of tough hydrogels.^[^ ^63^, ^64^, ^65^ ^]^

Hydrogen bonding is a short‐range electrostatic interaction between a hydrogen atom (covalently bound to an electronegative atom X), and a second electronegative atom Y, bearing a lone pair of electrons, thus, forming a hydrogen‐bonded dimer denoted as X–H⋅⋅⋅Y (Figure 6). Hydrogen bonding is essential in many biological systems, for example to stabilize 3D assemblies of nucleic acids and proteins.^[^ ^66^ ^]^ Hydrogen bonds are substantially weaker (2–15 kcal mol^−1^) than covalent bonds, and relatively weak compared to other non‐covalent interactions.^[^ ^30^ ^]^ Often, individual molecules are connected by more than one hydrogen bond, as in the case of the monomers acrylamide or acrylic acid. In both instances, each hydrogen is interacting with the complementary carbonyl oxygen forming dimers. This strategy has been adapted for a number of hydrogen bond motifs, for instance, the quadruple hydrogen bonding motif ureidopyrimidinone (UPy) with an acceptor–acceptor–donor–donor array, which has been used in hydrogels, too.^[^ ^67^, ^68^ ^]^ For the synthesis of tough hydrogels, however, most networks have exploited rather small monomers capable of i) straight‐forward (co)polymerization, ii) multiple hydrogen bond formation per monomer, and iii) synergistic strenghening through multivalent binding within the network. In this regard, the selected hydrogen bonding monomers include acrylamide (AAm),^[^ ^69^ ^]^ *N*,*N*'‐dimethylacrylamide (DMAAm) in combination with acrylic acid (AAc),^[^ ^70^, ^71^, ^72^, ^73^ ^]^ vinyl imidazole (VIm),^[^ ^74^, ^75^ ^]^ custom designed monomers, such as *N*‐acryloyl glycinamide (NAGA),^[^ ^76^, ^77^ ^]^ and *N*‐acryloylsemicarbazide (NASC),^[^ ^78^, ^79^ ^]^ as well as other hydrogen bond promoting systems.^[^ ^80^, ^81^, ^82^, ^83^ ^]^

Microphase separation refers to the spontaneous separation of (chemically) incompatible components in a system to form separated phases in the microscopic regions of the material (Figure 6). Historically, the phenomenon originates from block copolymers and polymer blends, where individual blocks pack into regular arrangements to maximize attractive interactions and to minimize repulsive ones, as well as the total free energy of the system. The phase‐separated domains can have different chemical or physical properties, such as refractive index, elasticity, or surface energy, offering interesting opportunities for the synthesis of materials.^[^ ^84^ ^]^ Besides the bulk phase, microphase separation has been observed in solutions, gels and even cells, as has been shown recently.^[^ ^85^ ^]^ For the sake of data representation in this review, we associate microcrystallization and salting‐out effects to the phenomenon of microphase separation.

The process of crystallization begins with the nucleation of a small crystal (or seed), which successively grows into microcrystalline domains at the expense of surrounding amorphous ones. While this process has been observed for (semi‐)crystalline, bulk polymers or strain‐crystallizing rubbers,^[^ ^86^ ^]^ it can also occur in hydrogels, as seen in those based on poly(vinyl alcohol) (PVA).^[^ ^87^ ^]^ On a molecular level, the PVA chains arrange into a well‐defined lattice structure (supported by hydrogen bonding) and are surrounded by an amorphous incompatible bulk phase that lacks any long‐range order.^[^ ^88^ ^]^ The crystalline regions can then act as physical cross‐links due to their increased rigidity, higher local mechanical stiffness, and the reversible packing. PVA is a prime example where such microcrystallites can be triggered via temperature to mediate gelation and has seen avid interest for the fabrication of tough hydrogels.^[^ ^89^, ^90^, ^91^, ^92^, ^93^, ^94^, ^95^, ^96^, ^97^, ^98^, ^99^ ^]^

Another type of microphase separation can occur through the salting‐out effect, which was originally observed in protein chemistry. When weakly polar solutes are dissolved in water, certain salts can deprive the solute from its hydration shell, reduce solute–solvent interactions, and solute solubility, which eventually leads to its precipitation. The salting‐out effect is more pronounced by anions and follows the so‐called Hofmeister series: CO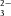 > SO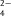 > S_2_O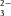 > H_2_PO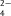 > F^−^ > Cl^−^ > Br^−^ ∼ NO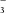 > I^−^ > ClO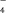 > SCN^−^.^[^ ^100^ ^]^ Analogous to proteins, well‐hydrated anions (e.g., SO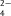 and CO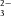) can polarize the water molecules in the hydration shell of synthetic polymers, destabilizing their surrounding hydrogen bonding network.^[^ ^101^ ^]^ When a hydrogel experiences salting‐out, polymer chains partially collapse rather than precipitate, resulting in microphase separated (sometimes crystalline) polymer clusters that act as physical cross‐links within the network.^[^ ^102^, ^103^ ^]^

Traditionally, microphase separation is associated with block copolymers that consist of at least two immiscible blocks (often hydrophilic and hydrophobic). In solution, microphase separation can be facilitated by adding surfactants that promote the micellization of hydrophobic entities in an aqueous bulk phase. Consequently, micellized hydrogels can be obtained through polymerization when i) micelles are loaded with hydrophobic monomers and ii) sufficient amounts of hydrophilic monomers are present in the aqueous bulk phase (Figure 6). Among the hydrophobic monomers, (meth)acrylates with aliphatic alkyl groups (e.g., stearyl, lauryl) have been frequently selected.^[^ ^104^, ^105^, ^106^, ^107^ ^]^ The interaction between these hydrophobic alkyl groups is on the order of 0.37 kcal mol^−1^ per –CH_2_– unit and therefore, can exert significant impact, once many thousands of molecules assemble.^[^ ^30^ ^]^ In the resulting hydrogel, the polymerized micellar aggregates can act as dynamic cross‐links as they deform and re‐assemble under loads providing the gel with viscoelastic properties. Micellization has therefore become a potent mechanism of energy dissipation contributing to the development of tough hydrogels.^[^ ^108^, ^109^, ^110^, ^111^ ^]^

Being well aware of the fact that micellization is also a result of hydrophobic interactions, we nonetheless distinguish between both categories to enable a clear arrangement of the data on tough hydrogels discussed in this review. Hence, active pre‐assembly of polymerizable building blocks and surfactants prior to their polymerization is categorized as micellization, while microphase separation in the absence of surfactants driven solely by hydrophobic interactions is classified as such (Figure 6). Respective examples discussed in this review often involve film‐casting and solvent‐exchange methods. First, block copolymers are homogeneously dissolved in an organic solvent followed by casting a polymer film in a mold. Upon drying of the film, hydrophobic interactions drive microphase separation of the block copolymers. When the dried film is immersed in water, residual organic solvents are removed giving rise to the final microphase‐separated tough hydrogel. Selected systems use hydrophobic monomers, such as butyl methacrylate (BuMA),^[^ ^112^ ^]^ octyl methacrylate (OMA),^[^ ^113^ ^]^ stearyl acrylate (SA),^[^ ^114^ ^]^ 2‐(N‐ethylperfluorooctanesulfonamido)ethyl acrylate (FOSA),^[^ ^115^ ^]^ phenyl acrylate (PhA),^[^ ^116^ ^]^ acrylonitrile (AN),^[^ ^117^, ^118^ ^]^ among other formulations.^[^ ^119^, ^120^ ^]^

Composite materials generally consist of individual constituents, each possessing distinct properties, which combined give rise to a new material with superior properties. Nanocomposite hydrogels comprise a bulk phase of water‐swollen polymer chains and at least one other phase of a solid materials with nanoscale morphology (often referred to as nanofillers, Figure 6). To form cross‐links that stabilize the network, polymer chains are either covalently or physically bound to the surface of the embedded nanostructures.^[^ ^121^ ^]^ In the first case, the nanoparticle surface is modified with chemical ligands that can either be copolymerized (grafting from) or reacted with/replaced by functional polymers forming stable bonds with the surface (grafting onto). In the second case, polymer chains are adsorbed to the nanomaterial surface through electrostatic or dispersion interactions. Haraguchi et al. pioneered the field of nanocomposite hydrogels by utilizing exfoliated clay nanosheets in combination with a surface‐adsorbing initiator. Once the polymerization was started, the clay‐polymer adsorption caused gelation of the system with each clay sheet acting as a giant cross‐linking point of higher functionality.^[^ ^122^ ^]^ Upon mechanical deformation, the reversible attachment of polymer chains together with re‐orientation of clay nanosheets synergistically enabled energy dissipation. This concept was carried forth and implemented into a variety of morphologies ranging from nanospheres or nanosheets to nanofibers. To obtain tough nanocomposite hydrogels, a variety of spherical nanoparticles (NP) has been exploited based on SiO_2_,^[^ ^123^, ^124^, ^125^, ^126^ ^]^ metal/metal–oxides,^[^ ^127^, ^128^, ^129^ ^]^ calcium phosphate (CaP),^[^ ^130^, ^131^, ^132^ ^]^ CaCO_3_,^[^ ^133^, ^134^ ^]^ biochar,^[^ ^135^ ^]^ or rubber microspheres.^[^ ^136^, ^137^, ^138^ ^]^ Nanosheet‐supported tough composite hydrogels have followed Haraguchi's example and used different forms of clay (Laponite).^[^ ^137^, ^139^, ^140^, ^141^ ^]^ Although they have not been yet explored in a fracture mechanical context, other nanosheet structures such as graphene or MXenes have seen growing use as nanofillers for hydrogels.^[^ ^142^, ^143^ ^]^ In addition to nanospheres and nanosheets, fibers (or fiber mats) have also been exploited to form tough (nano)composite hydrogels.^[^ ^144^, ^145^, ^146^, ^147^, ^148^ ^]^ To sum up, the sheer range of morphologies, dimensions, sizes combined with the various physical and chemical properties of chosen nanomaterials has made (nano)composite hydrogels a popular and successful area for the fabrication of energy‐dissipating tough hydrogels.

For the last category of energy dissipation, we chose the overarching term of self‐assembly. The respective systems are often driven by a combination of multiple short‐range interactions and therefore, cause complex self‐assembly processes, such as in helix formation, protein folding or the binding of guest to host molecules. Helix conformations are common structural elements of biopolymers (e.g., DNA α‐helix).^[^ ^149^ ^]^ Similarly, certain natural polymers, such as gelatin, gellan gum, and agarose, form helical structures that are stabilized by hydrogen bonding and/or ionic interactions (Figure 6). Gelatin is a degradation product of collagen, while agarose is a polysaccharide extracted from certain seaweed. Both are soluble in water and enter a helix‐to‐coil transition above temperatures of 40 °C. Upon cooling biopolymer solutions, gelation occurs due to the reformation of double helices resulting in thermoreversible networks.^[^ ^150^, ^151^, ^152^ ^]^

Many naturally occurring biological materials contain folded globular proteins, which can undergo reversible force‐induced unfolding, leading to large extension of the polypeptide chain and effective energy dissipation.^[^ ^153^ ^]^ In the case of the giant muscle protein titin, reversible unfolding helps to prevent damage to muscle tissue, for instance, due to overstretching. Similarly, elastin‐like proteins (ELP) mimic the rubber‐like stretchability of elastin, one of the most abundant proteins in the human body. Both cases have inspired the development of tough hydrogels through protein folding alike (Figure 6).^[^ ^154^, ^155^, ^156^ ^]^

Host–guest inclusion complexation occurs when a guest molecule penetrates the interior cavity of a larger, hollow host molecule, with the earliest investigated host molecules being crown ethers and cyclodextrins (CD).^[^ ^157^ ^]^ The latter are cyclic oligosaccharides with either 6 (α‐CD), 7 (β‐CD), or 8 (γ‐CD) glucose repeating units. Beside CDs, other macrocycles such as cucurbit[n]urils (CB[n]) or pillar[n]arenes have gained increasing interest in the material sciences.^[^ ^158^, ^159^ ^]^ CB[n] macrocycles consist of glycoluril units and have much more rigid cavities as compared to CDs, their portals being laced with carbonyl groups that enable strong binding with cationic guests. Compared to CDs, CB[n] host–guest interactions cover a broad range of binding affinities reaching values up to *K*_*a*_ = 10^17^ M^−1^.^[^ ^160^ ^]^ Consequently, they have been used in a variety of supramolecular hydrogels and although stiff and stretchable hydrogels were obtained, their fracture mechanical properties largely remained unknown.^[^ ^158^, ^161^ ^]^ Rather than classical host–guest inclusion complexation involving guest‐ and/or host‐functionalized polymers, the two discussed examples of tough hydrogels herein used CD host–guest self‐assembly to enable a unique network topology known as slide‐ring gels (Figure 6). In this scenario, CDs are threaded onto long polymer chains prior being cross‐linked to form a network with slidable cross‐links. The consequences of slidable cross‐links onto the material properties are fascinating and have created both a new subfield in the area, as well as a powerful energy dissipation mechanism for tough hydrogels.^[^ ^162^, ^163^ ^]^

## 4 Fracture Mechanical Properties of Tough Hydrogels

In the following part the mechanical properties of state‐of‐the‐art tough hydrogels are discussed in more detail. The tough hydrogels are classified and discussed according to their modes of energy dissipation, which have been previously defined (Figure 6): electrostatic interactions, microphase separation, composites, and self‐assembly. On account of the rich amount of reported materials, in selected cases, the subcategories are discussed separately (e.g., metal–ligand interactions, ionic interactions, etc.). Additionally, we highlight the water content of the presented data on tough hydrogels, as it substantially impacts the (fracture) mechanical properties.

### 4.1 Electrostatic Interactions

**Figure 7:**
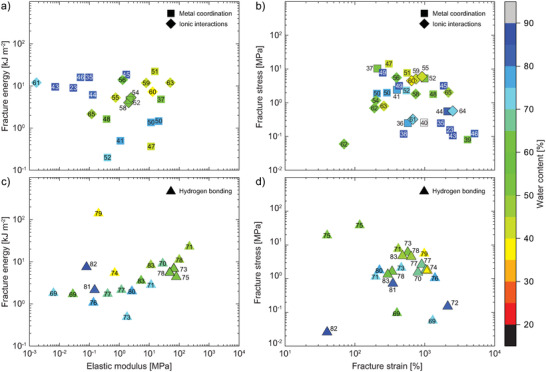
Tough hydrogels with energy dissipation driven by metal coordination (squares), ionic interactions (diamond), and hydrogen bonding (triangles) showing plots of fracture energy versus elastic modulus (a + c) and fracture stress versus fracture strain (b + d) with each datapoint featuring the corresponding citation number. Color grading corresponds to the water content of the respective hydrogel specimen, hollow symbols represent samples of unknown water content.

Metal coordination represents a powerful energy dissipation mechanism, as it features a large bandwidth of metal–ligand combinations, many of which are also abundant in nature (**Figure** **7**a,b). The metal coordination between divalent calcium ions and the biopolymer alginate (Alg) is a prime example for reversible cross‐linking; and as illustrated by Sun et al., paved the way toward tough and stretchable hydrogels.^[^ ^23^ ^]^ In this pioneering work, a Ca^2 +^ cross‐linked Alg network was combined with a second network made from acrylamide (AAm) covalently cross‐linked with *N*,*N'*‐methylenebis(acrylamide) (MBA). Albeit their elastic modulus was limited to *E* = 29kPa, the reported double network hydrogels were highly stretchable ($\varepsilon_{b}=2300\%$) and tough ($\Gamma =9{\mathrm{kJm}}^{-2}$) at a water content of 90%.^[^ ^23^ ^]^ Several studies on the mechanical properties of PAAm/Alg–Ca^2 +^ double networks emerged thereafter.^[^ ^35^, ^36^, ^38^, ^39^ ^]^ A notable improvement was achieved, when blends of short and long‐chain alginates were used as second network.^[^ ^35^ ^]^ Through incorporation of 33% short‐chain alginate, the fracture toughness of the double network rose to $\Gamma =16\;\mathrm{kJ}\;m^{-2}$ with an elastic modulus of *E* = 98kPa. Comparing these results to Alg–Ca^2 +^ single networks reveals the strength of the double network architecture. For example, in a recent study, where Alg–Ca^2 +^ single networks were generated via slow evaporation of diluted pre‐gel solutions, fracture energies were much lower ($\Gamma =0.5\;\mathrm{kJ}\;m^{-2}$), despite the gel sample being relatively stiff (*E* = 1 MPa at water content of 75%).^[^ ^41^ ^]^

Analogous to other metal–ligand complexes, Alg binds to various metals aside from Ca^2 +^, which was first explored by Mo̊rch et al. in polymer microbeads,^[^ ^164^ ^]^ and later tested in bulk hydrogels.^[^ ^165^ ^]^ Both studies indicated that metal–alginate binding affinities increase according to Ca^2 +^< Sr^2 +^< Ba^2 +^< Cd^2 +^< Cu^2 +^< Pb^2 +^< trivalent cations, which was also reflected by the hydrogel's mechanical properties. This knowledge was picked up by Liang et al, who introduced trivalent Fe^3 +^ cations to their double network.^[^ ^37^ ^]^ Additionally, the first network, which has been commonly made from a PAAm homopolymer, was adjusted to contain acrylic acid (AAc) groups. As a result, Fe^3 +^ ions interacted with the acid groups of both the P(AAm‐*stat*‐AAc) and the alginate network, interconnecting the two. This synergistic network stabilization significantly enhanced the elastic modulus (*E* = 24.6 MPa), leaving the fracture energy comparatively behind ($\Gamma =4.8\;\mathrm{kJ}\;m^{-2}$, water content 51%).^[^ ^37^ ^]^ Nonetheless, this method of cross‐linking also showed a distinct improvement to the fracture stress, which for this system is among the highest values reached (σ_b_ = 10.4 MPa).

Undoubtedly, the first reported PAAm/Alg–Ca^2 +^ double network inspired many more contributions, which further explored variations of network microstructure, cations, or initiator.^[^ ^40^, ^42^, ^43^, ^44^, ^45^, ^46^ ^]^ Notably, the polysaccharide carrageenan has become a popular replacement for alginate.^[^ ^43^, ^44^, ^45^, ^46^ ^]^ In the presence of cations, such as Mg^2 +^, Ca^2 +^, or K^+^, carrageenan (Carr) polymer strands can aggregate into helices.^[^ ^166^ ^]^ The aggregation with K^+^ in particular was successfully exploited to generate tough PAAm/κ‐Carr double networks. With a water content of 86%, Fei and co‐workers reached a fracture energy of $\Gamma =9.5\;\mathrm{kJ}\;m^{-2}$, albeit the stiffness remained limited (*E* = 7.2kPa).^[^ ^43^ ^]^ A different example by Li and co‐workers showed, how a similar system (82% water content) increased the stiffness to *E* = 130 kPa, yet at the expense of the fracture energy, which fell to $\Gamma =6.15\;\mathrm{kJ}\;m^{-2}$.^[^ ^44^ ^]^ Both examples show how an impressive stretchability (beyond 2000% before failure) often comes at the expense of the elastic modulus of the system. Adding gelatin into the formulation increased the stretchability even further, as it provided additional energy dissipation through helix formation at reduced temperatures. In this recent work, such extreme fracture strains ($\varepsilon_{b}=5170\%$) can be a major driving force to increase the fracture energy ($\Gamma =16.05\;\mathrm{kJ}\;m^{-2}$).^[^ ^46^ ^]^ The highest fracture energy of $\Gamma =18.5\;\mathrm{kJ}\;m^{-2}$ that has been demonstrated for a PAAm/κ‐Carr double network has been reported by Yu et al. This improvement was largely attributed to the change from K^+^ to Zr^4 +^, which enhanced the binding interactions between the κ‐Carr sulfate groups, tightening the helices. As a result, the much stiffer material reached an elastic modulus of *E* = 1.7 MPa, still resisting a remarkable elongation of $\varepsilon_{b}=1870\%$.^[^ ^45^ ^]^

Complementary to double networks, metal‐binding comonomers have been copolymerized directly into single networks to yield energy‐dissipating tough hydrogels.^[^ ^47^, ^48^, ^49^, ^50^, ^52^ ^]^ For instance, Xu et al. copolymerized vinyl diaminotriazine with vinyl imidazole (VIm) in the presence of small quantities of PEG diacrylate (PEGDA) as covalent cross‐linker. Diaminotriazine dimerizes via hydrogen bonding, while VIm units chelate Zn^2 +^ cations. Both displayed samples exhibited high stiffness (*E* = 20.6 and11.4 MPa at 76% water content), yet the fracture energies remain on the lower scale ($\Gamma =1.37-1.47\;\mathrm{kJ}\;m^{-2}$).^[^ ^50^ ^]^ Wang and co‐workers relied on Cu^2 +^ ions instead. First, they prepared a P(AAm‐*stat*‐VIm)‐based network, which was covalently cross‐linked using small amounts of MBA. By soaking this network in aqueous Cu^2 +^ solutions, the imidazole groups formed copper complexes, leading to a significant increase of both stiffness (*E* = 15.4 MPa) and fracture energy ($\Gamma =22.1\;\mathrm{kJ}\;m^{-2}$).^[^ ^51^ ^]^ The water content of this hydrogel was reduced to 44%, likely an adverse effect of the soaking process, which may have caused water expulsion from the network.

From the data presented throughout this last section, it becomes apparent that double networks represent potent scaffolds to increase fracture energies. Single networks with metal coordination have been reported with higher stiffnesses, however, in most cases their fracture energies are lower. Dual cross‐linking in a single network might be a strategy to alleviate this setback, as seen by the last example, where metal coordination is complemented with few covalent cross‐links in the same network.

Although hydrogels based on polyampholytes (polymers with both positive and negative charges) have been known since the early 1950s,^[^ ^167^, ^168^ ^]^ it was not until 2013 when they were used to prepare tough hydrogels.^[^ ^169^ ^]^ Since then, Gong and co‐workers have significantly contributed to polyampholyte hydrogels, where ionic attractions between oppositely charged comonomers led to tough, mechanically robust materials (Figure 7a,b).^[^ ^58^, ^59^, ^60^, ^61^, ^62^ ^]^ Several outstanding examples made use of sodium *p*‐styrenesulfonate comonomers (NaSS). The first work combined the negatively charged monomer with positively charged acryloyloxethyltrimethylammonium chloride (DMAEA‐Q).^[^ ^59^ ^]^ With a water content of 43%, these tough single networks achieved elastic modulus and fracture energy of *E* = 7.9MPa and $\Gamma =11.8\;\mathrm{kJ}\;m^{-2}$, respectively. Changing the positively charged monomer to 3‐(methacryloylamino)propyl‐trimethylammonium chloride (MPTC) had little effect onto the overall mechanical properties, unlike the addition of various PEG substrates as osmolytes.^[^ ^60^ ^]^ In the presence of PEG in different concentrations, the hydrogels underwent phase transition from the viscoelastic to the glassy state. For instance, using 30 wt% of PEG for the gel sample (overall water content of 39%), an elastic modulus and a fracture energy of *E* = 12.9 MPa and $\Gamma =7.3\;\mathrm{kJ}\;m^{-2}$ were obtained, respectively. The interactions between ionic groups are generally based on charge balance and therefore requires each positively charged residue to be compensated by a negatively charged one. In a recent example, Gong and co‐workers presented a dual cross‐linked network (covalent & ionic), where postively charged monomers are combined with sulfobetaine methacrylate.^[^ ^61^ ^]^ The resulting tough gels had a water content of 70% and while they showed good fracture properties ($\Gamma =12\;\mathrm{kJ}\;m^{-2}$), the stiffness was determined to be rather low (*E* = 1.7kPa). The latter could be a result of charge imbalances through use of the zwitterionic sulfobetaine monomer, which natively carries both charges in the first place.

Regarding natural polymers, chitosan (CS) has played an increasing role in the development of ionically driven, tough hydrogels (Figure 7a,b). Various reports combined CS with sodium phytate,^[^ ^54^, ^55^ ^]^ citrate,^[^ ^56^ ^]^ polypyrrole,^[^ ^63^ ^]^ or AAc copolymers.^[^ ^64^, ^65^ ^]^ The highest stiffness was observed for the tough hydrogel made from PAAm/CS–polypyrrole (*E* = 50.1 MPa), where a PAAm network was combined with interpenetrating CS chains, Fe^3 +^ ions, and in situ forming rigid polypyrrole chains. The authors reported a fracture energy of $\Gamma =12\;\mathrm{kJ}\;m^{-2}$ at a stated water content of 75%; this figure is likely lower as neither the amounts of polypyrrole (20%) nor FeCl_3_ are factored in.^[^ ^63^ ^]^ The highest fracture energy among CS‐based systems was measured, when trivalent citrate anions (Cit^3 −^) were used as ionic cross‐linker in a PAAm/CS–Cit^3 −^ double network. Mechanical properties amounted to *E* = 1.3 MPa and $\Gamma =14\;\mathrm{kJ}\;m^{-2}$ at a moderate water content of 56%.^[^ ^56^ ^]^ Lastly, the work by Fan et al. should be mentioned, which in addition to ionic interactions introduced hydrophobic domains by use of an aliphatic catechol‐functional monomer. While the fracture toughness is showing an average value of $\Gamma =6.6\;\mathrm{kJ}\;m^{-2}$, the fracture strain exceeds 2500% (the elastic modulus was not reported).^[^ ^64^ ^]^ Generally, tough polyampholyte hydrogels require higher polymer fractions to maintain strong ionic interactions, which often comes at the expense of the water content in these gels (in the range of 40–60%). Similar to PAAm/Alg or PAAm/Carr double networks, PAAm/CS–Cit^3 −^ double networks achieve good fracture energies along with remarkable stretchabilities.

Hydrogen bonds can be a powerful motif to enhance reversible interactions between polymer chains and hence, dissipate energy (Figure 7c,d). Certainly, PAAm is a polymer with extensive hydrogen bonding where amide N–H groups act as donors to the amide C = O hydrogen acceptors. This was investigated in an article by Ballance et al, where PAAm was loosely covalently cross‐linked.^[^ ^69^ ^]^ With a water content of 71%, stiffness and fracture energy remained relatively low (*E* = 6.3 − 28 kPa, $\Gamma =1.75-1.85\;\mathrm{kJ}\;m^{-2}$). Contrary to AAm, *N*,*N*'‐dimethylacrylamide (DMAAm) exhibits strong hydrogen bond accepting properties. In 2015, Sheiko and co‐workers reported a combination of DMAAm and methacrylic acid (MAAc).^[^ ^70^ ^]^ In addition to the hydrogen bond donating properties of MAAc, its α‐methyl group promotes hydrophobic interactions between polymer backbones throughout the polymerization. As a result, domains with a high concentration of hydrogen bonds are formed that act as energy‐dissipating clusters. With this strategy, the hydrogels reached a stiffness and a fracture energy of *E* = 28 MPa and $\Gamma =9.3\;\mathrm{kJ}\;m^{-2}$, respectively at a water content of 67%.^[^ ^70^ ^]^ This concept was later adapted by Wang et al., who used MAAc in conjunction with methacrylamide (MAAm). With water contents ranging from 70–46%, they covered an impressive range of elastic moduli and fracture energies of *E* = 11.5 − 217.3 MPa and $\Gamma =2.9-23.5\;\mathrm{kJ}\;m^{-2}$, respectively.^[^ ^71^ ^]^ Other monomers were introduced, such as phenylalanyl methacrylate (reaching $\varepsilon_{b}=2000\%$)^[^ ^72^ ^]^ or *N*‐pyridyl acrylamide (NPyAAm).^[^ ^73^ ^]^ Beside its use in metal–ligand interactions, VIm is also capable of forming hydrogen bonds. As such, it was used as hydrogen bond acceptor in combination with AAc,^[^ ^74^ ^]^ and MAAc.^[^ ^75^ ^]^ The fracture energies of the representative samples were in a similar range ($\Gamma =5.6-4.6\;\mathrm{kJ}\;m^{-2}$), while the stiffness of poly(VIm‐*stat*‐MAAc) was approximately two orders of magnitude higher (reaching *E* = 80 MPa). The latter also exhibited strongly improved fracture stress (σ_*b*_ < 40 MPa), which came at the expense of the failure strain ($\varepsilon_{b}=100\%$).

Recently, various new monomers have been designed that exhibit multiple acceptor/donor sites per molecule thus, multiplying the amount of hydrogen bonds throughout the network. Liu and co‐workers introduced *N*‐acryloyl glycinamide (NAGA) and also *N*‐acryloylsemicarbazide (NASC), which can engage in 3–4 hydrogen bonds at the same time.^[^ ^76^, ^77^, ^78^ ^]^ Remarkably, when just NASC is homopolymerized in different concentrations, an increase of monomer concentration from 15 to 30 v/v% leads to a doubling in both stiffness (*E* = 48.4 − 100.3 MPa) and fracture energy ($\Gamma =5.65-11.4\;\mathrm{kJ}\;m^{-2}$), while the water content is reduced from 61% to 46%.^[^ ^78^ ^]^ The same monomer (NASC) was later copolymerized in various ratios with AAc to yield the highest fracture energy within the category of electrostatic interactions. In the mentioned work by Wu et al, a fracture toughness of $\Gamma =144\;\mathrm{kJ}\;m^{-2}$ was reported, markedly coming at the expense of both stiffness (*E* = 0.2MPa) and water content of the hydrogel (36%).^[^ ^79^ ^]^ The fact that both preceding examples are single networks further emphasizes the capacity of hydrogen bonding as a potent mode of energy dissipation.

Other hydrogen bond assisted tough hydrogels combined hydrophilic polyurethanes (PU) with lignin,^[^ ^80^ ^]^ tannic acid,^[^ ^81^ ^]^ or used gellan gum and gelatin as hydrogen bond donors.^[^ ^82^, ^83^ ^]^ Overall, the majority of samples with elastic moduli exceeding several MPa are in line with reduced water contents in the range of 40%–55%. Additionally, it was shown that clever design of hydrogen bonding networks could substantially push the boundaries of both elastic moduli and fracture energies. Analogous to ionically‐driven tough hydrogels, the multiplication of hydrogen bonds within the gel is key to increase stiffness and fracture toughness, which is in turn tied to higher polymer fractions and reduced water contents. Nonetheless, the gels with the most remarkable (fracture) mechanical properties in this category are single networks.

### 4.2 Microphase Separation

**Figure 8:**
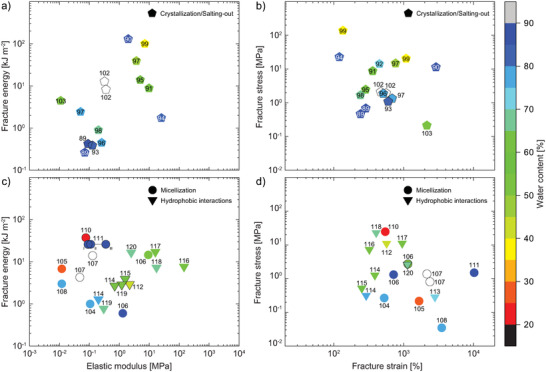
Tough hydrogels with energy dissipation driven by crystallization/salting‐out (pentagon), micellization (spheres) and hydrophobic interactions (top‐down triangles) showing plots of fracture energy versus elastic modulus (a + c) and fracture stress versus fracture strain (b + d) with each datapoint featuring the corresponding citation number. Color grading corresponds to the water content of the respective hydrogel specimen, hollow symbols represent samples of unknown water content.

Microcrystallization is the process of polymer chains forming submicrometer‐sized crystallites, representing a microphase‐separated domain surrounded by amorphous polymer chains (**Figure** **8**c,d). PVA is an attractive candidate for the formation of hydrogels via crystallization.^[^ ^87^, ^88^ ^]^ During the process of freeze‐thawing, the polymer solution is frozen at degrees below 0 °C to grow the crystallites followed by thawing at room temperature. With incremental freeze‐thaw cycles, the hydrogel's mechanical properties are enhanced together with the overall crystallinity (saturating toward 35%).^[^ ^170^ ^]^ It is hence unsurprising that in various cases PVA has been exploited for the synthesis of tough hydrogels, especially given its non‐toxic and biocompatible nature (Figure 8a,b).^[^ ^89^, ^90^, ^91^, ^92^, ^93^, ^94^ ^]^

In a recent work, the method of directional freeze‐thawing was proposed to create tough hydrogels with anisotropic morphology. As opposed to freezing the hydrogel precursor solution at once, Zhang et al. prepared a mold that was lowered at a constant velocity into a bath of liquid nitrogen (much like a dip‐coating approach), causing the freezing process to occur in a directional manner. A PVA crystallinity of 55% was achieved through this procedure with SEM micrographs showing the oriented pore morphology of the hydrogels. Despite the hydrogel's anisotropy, selective parallel and orthogonal mechanical testing revealed only mild differences (*E*_∥/⊥_ = 70/90 kPa, $\Gamma_{∥/⊥}=0.27/0.43\;\mathrm{kJ}\;m^{-2}$).^[^ ^89^ ^]^ In a work by Hao et al., PVA was combined with hyaluronic acid (HA) and collagen to form a semi‐interpenetrating network (SIPN, since only the PVA is cross‐linked). The resulting gels showed a greatly improved stiffness (*E* = 25.4 MPa) at a high water content of 80%, yet a fracture energy of $\Gamma =1.27\;\mathrm{kJ}\;m^{-2}$ was measured.^[^ ^94^ ^]^

The microcrystallization of PVA successfully produced a number of double networks, such as PVA/PAAm,^[^ ^95^ ^]^ PVA/Alg–Ca^2 +^,^[^ ^96^, ^97^ ^]^ PVA/Pectin–Ca^2 +^,^[^ ^98^ ^]^ or PVA/PAAc–Fe^3 +^ (Figure 8a,b).^[^ ^99^ ^]^ The last mentioned report by Wang and co‐workers used a second network of PAAc in combination with Fe^3 +^ ions, which promoted metal–ligand interactions in both the PAAc and the PVA network interconnecting the two. This enabled excellent mechanical and fracture properties (*E* = 7.1 MPa, $\Gamma =101\;\mathrm{kJ}\;m^{-2}$), at the same time maintaining excellent strength and ductility of the hydrogel (σ_b_ = 20.7 MPa, $\varepsilon_{b}=1080\%$).^[^ ^99^ ^]^ Moreover, when the sample was cold‐drawn to orient the polymer chains, an elastic modulus of up to *E* = 100 MPa was measured with the fracture energy sadly not being reported for this sample. Likely, both the Fe^3 +^ as cross‐linker and the reduced water content of 36% contribute to the mechanical properties and it remains difficult to decouple the two.

In 2021, He and co‐workers indroduced an effective approach to further boost the mechanical properties of PVA‐based single networks.^[^ ^90^ ^]^ As opposed to Zhang et al.,^[^ ^89^ ^]^ the authors used directional freeze‐thawing at slower speed and higher temperature (–80 °C), which was followed by an additional salting‐out post‐processing step. The salting‐out was achieved through immersion of the gel in an aqueous citrate solution (1.5 m) and created additional crystalline clusters within the hydrogel matrix. As a result, the tough hydrogel exhibited a stiffness of *E* = 1.98 MPa, while reaching a fracture energy of $\Gamma =131\;\mathrm{kJ}\;m^{-2}$ and upholding an excellent stretchability (2900%).^[^ ^90^ ^]^ The water content was reported as 90% and should be carefully noted as it refers to the gel's water content prior to the salting‐out step. We believe that immersion in the citrate solution may result in both increase of the solid content inside the gel, as well as shrinkage due to the salting‐out effect. Nonetheless, this example nicely demonstrates, how a synergy between two interactions can result in hierarchical microstructure and significant improvements in (fracture) mechanical properties.

Only two other examples actively exploit the salting‐out effect and both cases use CS (Figure 8a,b).^[^ ^102^, ^103^ ^]^ In a so‐called “soak n' boost” strategy, SIPNs made from covalently cross‐linked PAAm and short‐chain CS, are soaked for 20 min in either alkaline 1 m NaOH or saturated NaCl solutions. The former is believed to produce microcrystalline domains transforming the SIPN into a stiff and tough double network ($\Gamma =12.9\;\mathrm{kJ}\;m^{-2}$, *E* = 0.319 MPa). In the latter case, a classic salting‐out effect is achieved and while this double network is slightly stiffer (*E* = 0.358 MPa), the fracture energy is lowered ($\Gamma =8.3\;\mathrm{kJ}\;m^{-2}$).^[^ ^102^ ^]^ The water content following the soaking step, however, was not determined and is expected to change due to swelling/shrinking. Overall, tough hydrogels obtained through microcrystallization managed to reach new heights in fracture energies and stiffness, while exhibiting moderate water contents.

As seen above, PVA or CS microphase separation is actively induced through a temperature change or soaking. In contrast, the formation of nano‐sized micelles can occur spontaneously and has paved the way toward energy‐dissipating tough hydrogels via reversible micelle aggregation upon the application of stress (Figure 8c,d). Most commonly, aliphatic monomers, such as stearyl (meth)acrylate (SMA/SA) or lauryl methacrylate (LMA) are combined with a surfactant (e.g., sodium dodecyl sulfate (SDS) or cetyltrimethylammonium bromide (CTAB)) in a pre‐assembly stage. The formed micelles are complemented by hydrophilic monomers in the surrounding aqueous solution, which later form the bulk network. Reported tough hydrogels involve P(AAm‐*stat*‐SMA)/SDS,^[^ ^104^, ^105^ ^]^ P(AAc‐*stat*‐SA)/CTAB,^[^ ^106^ ^]^ P(AAc‐*stat*‐LMA)/CTAB,^[^ ^107^ ^]^ among others.^[^ ^108^, ^109^, ^110^, ^111^ ^]^

One notable example was published by Liu and co‐workers, who polymerized CTAB‐stabilized, SA‐containing micelles in the presence of an aqueous MAAc‐rich bulk phase to obtain a first network. Immersion of this first network in a solution of AAm followed by photopolymerization produced a tough SIPN. The system was shown to be widely tunable, as can be witnessed by the two datapoints we report, with one gel exhibiting a water content of 90%, the other 54%.^[^ ^106^ ^]^ The reduction of the water content (resulting from an increase in CTAB concentration) improved all four key mechanical properties: Stiffness (*E* = 1.31 to 9.25 MPa), fracture toughness ($\Gamma =0.6\;\mathrm{to}\;14.6\;\mathrm{kJ}\;m^{-2}$), fracture stress (σ_b_ = 1.32 to 2.73 MPa) and fracture strain ($\varepsilon_{b}=717\;\text{to}\;1149\%$), certainly representing a rare occurence.^[^ ^106^ ^]^

When the hydrophobe‐to‐surfactant ratio is sufficiently high, a micellar solution can transition to an oil‐in‐water emulsion. In a fitting example, a methacrylate‐telechelic, amphiphilic PU was used as reactive emulsifier to stabilize oil droplets that contained acrylonitrile (AN) in the presence of an aqueous, AAm‐rich bulk phase.^[^ ^110^ ^]^ Upon polymerization, a dual cross‐linked network was obtained with a covalent AAm network and hydrophobic spherical aggregates (120 nm) made from PAN as physical cross‐links. The tough hydrogel shows high fracture energy ($\Gamma =37.2\;\mathrm{kJ}\;m^{-2}$) and fracture strength (σ_b_ = 24.7 MPa), yet the stiffness reaches only *E* = 76 kPa.^[^ ^110^ ^]^ Further to this, the bespoke gel sample is closer to the nature of an elastomer, since its water content is as low as 23%.

Wu and co‐workers proposed a similar strategy, using divinyl benzene (DVB) as micellar cross‐linker in a PAAm matrix.^[^ ^111^ ^]^ The final tough hydrogels reached a fracture toughness of $\Gamma =26\;\mathrm{kJ}\;m^{-2}$ at a much improved water content of 86%. During the tensile test, an admirable stretchability of 10200% was reached without sample failure. In addition, three different stages of deformation were observed during the tensile test, giving rise to three elastic moduli. The tensile behavior in stage I (*E* = 90 kPa, ε ⩽ 8700%) was attributed to the dynamic physical interactions of the hydrophobic DVB aggregates paired with the unfolding and alignment of the PAAm chains. Similar to amorphous polymers, in stage II the hydrogel experienced a sudden increase in stress followed by a yield point (the elastic modulus rose to *E* = 111 kPa, ε ⩽ 9400%). In the final stage III, the gel experienced severe strain‐hardening with a modulus of *E* = 357 kPa (ε ⩽ 10200%), likely due to a tension shift from the fully aligned PAAm chains onto the cross‐linked DVB clusters.

In the absence of surfactants, certain molecules can directly aggregate via hydrophobic interactions to form energy‐dissipating clusters within the hydrophilic network (Figure 8c,d). Within this category, two double networks are reported, one made from PAAm and the microphase‐separating block copolymer P(BuMA‐*b*‐AAc‐*b*‐BuMA),^[^ ^112^ ^]^ the other combining xanthan gum–Ca^2 +^ and P(AAc‐*stat*‐OMA).^[^ ^113^ ^]^ Beside the previous ones, various single networks have been reported involving PU/P(AAc‐*stat*‐SA),^[^ ^114^ ^]^ P(NIPAAm‐*stat*‐FOSA),^[^ ^115^ ^]^ P(AAm‐*stat*‐PhA),^[^ ^116^ ^]^ as well as different AN‐based copolymers.^[^ ^117^, ^118^ ^]^ Additionally, one formulation consists of a polyether‐based PU,^[^ ^119^ ^]^ a second one simply combines PDMAAm with hydrophobic lignin.^[^ ^120^ ^]^ With a slightly higher water content than^[^ ^106^ ^]^ (62% vs. 54%), Feng, Liu, and co‐workers used a P(AN‐*stat*‐AAm) network, cross‐linked with a 3 kDa PEG dimethacrylate (PEGDMA). The authors reported an elastic modulus of *E* = 16.0 MPa and a fracture toughness of $\Gamma =16.6\;\mathrm{kJ}\;m^{-2}$.^[^ ^117^ ^]^ When hydrophobic and metal–ligand interactions were combined through use of a P(AN‐stat‐AAc) copolymer and Zn^2 +^ ions, a similar competitive elastic modulus was observed (*E* = 17.4 MPa).^[^ ^118^ ^]^ The strong hydrogel (σ_b_ = 21.9 MPa) exhibited a fracture toughness of $\Gamma =7.1\;\mathrm{kJ}\;m^{-2}$ at a water content of 68%.^[^ ^118^ ^]^

The stiffest hydrogel through hydrophobic interactions was created by copolymerization of AAm, phenyl acrylate (PhA) and varying amounts of MBA. The solvent of the resulting P(PhA‐*stat*‐AAm) network was changed from DMSO to water for mechanical testing. With a water content of 60% and a fracture energy of $\Gamma =7.45\;\mathrm{kJ}\;m^{-2}$, the elastic modulus reached a value of *E* = 145 MPa.^[^ ^116^ ^]^

Interestingly, within this category most of the described single networks outperform the two respective double network hydrogels. This demonstrates, how collective hydrophobic interactions can be a potent energy dissipation pathway, generating good fracture energies along with high stiffness values. More generally, the above data also confirms the previously observed trend that decreasing water content correlates with increasing stiffness. Nonetheless, some examples manage to reconcile the two; something that otherwise can be conflicting, as high moduli require the chain length between cross‐links to be short.^[^ ^171^ ^]^

### 4.3 Composites

**Figure 9:**
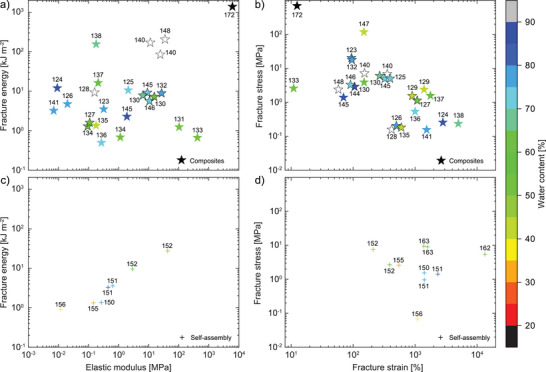
Tough hydrogels with energy dissipation driven through (nano)composite structures (stars) and self‐assembly processes (crosses) showing plots of fracture energy versus elastic modulus (a + c) and fracture stress versus fracture strain (b + d) with each datapoint featuring the corresponding citation number. Color grading corresponds to the water content of the respective hydrogel specimen, hollow symbols represent samples of unknown water content.

Tough (nano)composite hydrogels were grouped according to their morphology into hydrogels using (nano)spheres, nanosheets, or (nano)fibers (**Figure** **9**a,b). SiO_2_ nanospheres were employed in several studies,^[^ ^123^, ^124^, ^125^, ^126^ ^]^ while isolated cases employed Ag,^[^ ^127^ ^]^ or Fe_3_O_4_ NPs.^[^ ^128^ ^]^ In an example by Qiu, Wang and co‐workers, vinyl‐functional SiO_2_ NPs are first decorated with vinyl‐functional chondroitin sulfate polymers. Next, the decorated SiO_2_ NPs were copolymerized with AAm and small quantities of MBA (first network) in the presence of agar, which upon cooling formed the second network. At a water content of 77%, a strong double network was obtained (σ_b_ = 20.4 MPa), whose fracture energy and stiffness unexpectedly lacked behind ($\Gamma =3.544\;\mathrm{kJ}\;m^{-2}$, *E* = 0.321 MPa).^[^ ^123^ ^]^ Shi et al. copolymerized a solution containing vinyl‐functional SiO_2_ NPs, AAm, and SMA in the presence of SDS as surfactant. The resulting polymer network is covalently cross‐linked with the SiO_2_ NPs and dissipates energy via the micellar aggregates of the SMA. The fracture energy of the bespoke hydrogel reached a value of $\Gamma =12.1\;\mathrm{kJ}\;m^{-2}$ despite the rather low elastic modulus of *E* = 9.2 kPa.^[^ ^124^ ^]^ The latter was key to enhance the gel's stretchability, which amounted to $\varepsilon_{b}=2820\%$ at a water content of 90%. In a study by Guo et al., a PVA network is first established via freeze–thaw cycles, followed by soaking in sodium silicate solutions to fabricate robust dual cross‐linked networks. Their toughest hydrogel reached a fracture energy of $\Gamma =10.7\;\mathrm{kJ}\;m^{-2}$ and an elastic modulus of *E* = 2.1 MPa at a water content of 72%.^[^ ^125^ ^]^

An increasingly popular strategy to reinforce hydrogels has been the in situ mineralization of calcium phosphate (CaP),^[^ ^130^, ^131^, ^132^ ^]^ as well as CaCO_3_.^[^ ^133^, ^134^ ^]^ In 2020, Yu et al. presented a film‐casting technique to generate robust tough hydrogels consisting of PVA, NaAlg, and CaP. During the drying of the precursor film, CaP oligomers formed and transformed into hydroxy apatite nanocrystals through the interactions with surrounding PVA and Alg chains. The mineralization process produced a strong gel (σ_b_ = 17.84 MPa) and improved both the fracture and mechanical properties ($\Gamma =8.97\;\mathrm{kJ}\;m^{-2}$, *E* = 26.93 MPa), while the water content was maintained at 75%.^[^ ^132^ ^]^ Notably, while this gel had nearly identical failure stress and strain (σ_b_ = 17.8 MPa, $\varepsilon_{b}=96\%$) as the double network made from chondroitin sulfate/SiO_2_/agar,^[^ ^123^ ^]^ its elastic modulus is 90‐fold higher. On other occasions, tough hydrogels were supplemented with metal organic frameworks (MOF),^[^ ^129^ ^]^ or biochars NPs.^[^ ^135^ ^]^

As opposed to hard sphericles particles, soft polymer microspheres have been used to create strong and tough composite hydrogels.^[^ ^136^, ^137^, ^138^ ^]^ Tang et al. used sodium 2‐acrylamido‐2‐methyl‐1‐propanesulfonate (AMPS) in the presence of AAm and MBA to create a hydrogel sheet, which was subsequently freeze‐dried and extensively ground to yield a microgel powder. The final hydrogel was fabricated using a formulation consisting of AAm/MBA, clay and magnetic NdFeB nanoparticles, as well as various amounts of the ground microgel particles. While the NdFeB content slightly improved the overall strength and fracture energy of the gel, major stress–strain enhancements stemmed from the incorporation of the PAMPS microspheres. The optimimum formulation consisted of 2 wt% microgels and 10 wt% NdFeB NPs with the fracture toughness and elastic modulus reaching values of $\Gamma =16.4\;\mathrm{kJ}\;m^{-2}$ and *E* = 0.21MPa, while exhibiting moderate failure stress at good strain values (σ_b_ = 1.58 MPa and $\varepsilon_{b}=1770\%$).^[^ ^137^ ^]^ PAMPS microgels were also used in a report by Yan and co‐workers, except emulsion polymerization was used for their preparation. Using a water content of 67% and microgels (cured with 3.6 mol% cross‐linker) generated an extremely stretchable ($\varepsilon_{b}=5000\%$) hydrogel, which was capable of dissipating extensive amounts of energy ($\Gamma =157\;\mathrm{kJ}\;m^{-2}$).^[^ ^138^ ^]^ Comparatively, its stiffness (*E* = 0.18 MPa) and failure stress (σ_b_ = 0.237 MPa) lacked considerably behind.

Tough hydrogels reinforced by nanosheets have primarily used clay (Laponite) (Figure 9a,b).^[^ ^137^, ^139^, ^140^, ^141^ ^]^ In one notable report, a physical gel containing clay and a copolymer based on oligoethyleneglycol methacrylate (OEGMA) and NIPAAm was rapidly extruded into a fiber. Next, the authors immersed this gel fiber in aniline (ANI) solutions in the presence of phytic acid and a thermoinitiator to form the second network. The final double network fibers were swollen in water, however, the final water content was not stated. The combination of the soft non‐covalent nanocomposite network and the rigid PANI (cross‐linked with negatively charged phytic acid) brought forward a unique set of mechanical properties. Increasing the ANI immersion time from 6 to 8 min prior to the polymerization of the second network caused a significant change in fracture energy and stiffness ($\Gamma =172\;\text{to}\;85.7\;\mathrm{kJ}\;m^{-2}$, *E* = 11.4 to 24.4 MPa).^[^ ^140^ ^]^ In addition, the failure strain was approximately halved ($\varepsilon_{b}=356\;\text{to}\;153\%$), while the gel's strength was not affected (σ_b_ = 6.9 to 7.2 MPa).

Finally, several (nano)fiber composites were reported, where the hydrogel matrix was directly supplemented with gelatin fiber mats,^[^ ^144^ ^]^ aramid fibers,^[^ ^145^, ^146^, ^147^ ^]^ glass fibers,^[^ ^148^ ^]^ or silver nanowires (Figure 9a,b).^[^ ^146^ ^]^ In a work by Kotov and co‐workers, a dispersion of aramid nanofibers was combined with a solution of PVA to fabricate tough hydrogels via solvent exchange from DMSO to water. Two samples were selected with a PVA content of 8 and 30 wt%. The respective drop in water content (92% to 70%) increased the fracture energy and the elastic modulus, accordingly ($\Gamma =2.3\;\;\text{to}\;9.2\;\mathrm{kJ}\;m^{-2}$ and *E* = 1.9 to 9.1 MPa).^[^ ^145^ ^]^

Instead of using nanofibers, He et al. fixated an entire polyaramid fiber mat inside a mold before injecting a pre‐gel solution of AAc and PVA. Light‐controlled polymerization of the mold followed by freeze‐thaw cycles yielded the hydrogel, which was then immersed in a saline solution until the equilibrium swelling state was reached. By immersing the sample in seawater instead of LiCl, the hydrogel specimen reached a fracture toughness of $\Gamma =195.8\;\mathrm{kJ}\;m^{-2}$, the elastic modulus was not reported.^[^ ^147^ ^]^ Since the final synthetic step involved the immersion in salt solution, water contents varied and were stated to be in between 40–70%.

As an alternative to polyaramid fibers, glass fibers have been used as mechanical enhancer for tough hydrogels. In a study by Yang and co‐workers, PAAm/Alg–Ca^2 +^ double networks were supplemented with vinyl‐functional glass fabrics. First, the glass fabric was fixated in the center of the mold, followed by injection of the PAAm/Alg precursor solution and polymerization. Lastly, the composite gel was soaked in a 0.3 m CaCl_2_ solution, leaving the final water content of the gel undisclosed. The latter showed strongly enhanced fracture energy and elastic modulus of $\Gamma =206.7\;\mathrm{kJ}\;m^{-2}$ and *E* = 35 MPa, some of the highest combined values to date.^[^ ^148^ ^]^ The literature example that follows is primarily of instructive nature as it involves an elastomer with a water content of 0%. In the mentioned work by Gong and co‐workers, a glass fiber fabric was embedded into a rubbery matrix with soft and hard segments to yield a strong viscoelastomer (σ_b_ = 700 MPa). While its stretchability was reduced to a minimum ($\varepsilon_{b}=12.5\%$), the elastomeric material exhibited extreme values for fracture energy and elastic modulus ($\Gamma =1400\;\mathrm{kJ}\;m^{-2}$, *E* = 6120 MPa).^[^ ^172^ ^]^ Conclusively, (nano)fibers may greatly enhance the fracture mechanics of tough hydrogels, however, their ductility often suffers greatly, especially when macroscopic fiber mats are inserted.

### 4.4 Self‐Assembly

In the final section, tough hydrogels driven by self‐assembly are discussed (Figure 9c,d). While the formation of helices is often solely associated to strands of DNA, it also occurs in biopolymers such as agar,^[^ ^150^, ^151^ ^]^ gelatin or gellan gum.^[^ ^152^ ^]^ A noteworthy example combined both gelatin and gellan gum in a double network with water contents ranging from 57% to 49%. This reduction was caused by a change from ammonium sulfate to sodium sulfate as immersion salt; the sulfate acted as salting‐out agent, while the sodium was able to additionally promote helix‐to‐helix attractions. Thus, the resulting fracture energy and elastic modulus of the double network were significantly enhanced ($\Gamma =9.6\;\;\text{to}\;27.7\;\mathrm{kJ}\;m^{-2}$ and *E* = 2.9 to 42.6 MPa).^[^ ^152^ ^]^

When it comes to protein folding, only three studies have been published to date, which look into the fracture mechanics of the resulting hydrogel materials.^[^ ^154^, ^155^, ^156^ ^]^ A promising source of energy dissipation have been introduced by elastomeric proteins, which function as molecular springs and unfold once a stress is applied.^[^ ^173^ ^]^ This mechanical unfolding can be observed in smaller elastin‐like polypeptides, as well as in larger proteins (e.g., G8) and hence, was exploited for the synthesis of tough hydrogels alike ($\Gamma =0.9-1.33\mathrm{kJ}m^{-2}$).^[^ ^155^, ^156^ ^]^

Self‐assembly between host and guest molecules is a long‐known phenomenon and has been exploited in a multitude of materials, yet studies on the fracture mechanics of host–guest mediated hydrogels remain scarce. As a recent innovation, host–guest interactions have been specifically used to furnish networks with slidable cross‐links. Slidable cross‐links are neither covalent nor non‐covalent and can be described as an interlocked topological feature, which has been extensively studied by Ito and co‐workers. In their reports, macrocyclic cyclodextrin (CD) host molecules were threaded onto PEG polymer chains (guests) prior to chemically linking threaded CD molecules together.^[^ ^174^, ^175^, ^176^ ^]^ Upon the application of stress to the network, energy can be dissipated by the sliding motion of individual cross‐links along the polymer backbone.^[^ ^177^ ^]^ Recently, Ito and co‐workers constructed networks based on this slide‐ring topology and benchmarked them with comparable networks, where the cross‐links were fixed. The control sample was extended to $\varepsilon_{b}=310\%$ (σ_b_ = 0.45 MPa), while the corresponding slide‐ring gel reached an unmatched failure strain of $\varepsilon_{b}=13400\%$ representing the highest reported value for a tough hydrogel at the time of writing (σ_b_ = 5.5 MPa, water content: 62%).^[^ ^162^ ^]^ The same gel showed a fracture energy of $\Gamma =3.6\;\mathrm{kJ}\;m^{-2}$ (vs. $\Gamma =0.24\;\mathrm{kJ}\;m^{-2}$ for the control) and the elastic modulus was not reported. The concept of slide‐ring gels was slightly adapted by Chen et al. who produced gels that consisted of a network of CD molecules (connected with epichlorhydrin). This gel was then soaked in a solution containing aliphatic C12 bis(methacrylate), which could enter the CD cavity to form host–guest assemblies. Subsequently, chosen amounts of photoinitiator and 2‐hydroxyethyl acrylate (HEA) were added to this host–guest solution to form a tough and stretchable hydrogel upon polymerization. The double network hydrogel demonstrated good strength (σ_b_ = 9.5 MPa) and stretchability ($\varepsilon_{b}=1385\%$), combined with a fracture energy of $\Gamma =66.3\;\mathrm{kJ}\;m^{-2}$ (elastic modulus was not reported).^[^ ^163^ ^]^

**Figure 10:**
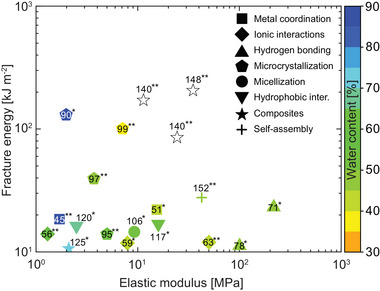
Energy‐dissipating tough hydrogels that unite high fracture energies with high elastic moduli. Single and double networks are marked with a single (*) and double asterisk (**), respectively.

To summarize, it remains difficult to unite excellent fracture energies with high elastic moduli in tough hydrogels. Nonetheless, recent years have produced unique combinations of interactions and materials to yield tough hydrogels with extreme mechanical properties, which are represented throughout all subcategories we have discussed (**Figure** **10**). As of today, composite materials have reached some of the highest values ($\Gamma ≳100\;\mathrm{kJ}\;m^{-2}$) by use of clay nanocomposite double networks (P(OEGMA‐*stat*‐NiPAAm)/PANI)^[^ ^140^ ^]^ or glass fiber‐reinforced PAAm/Alg double networks (although their water content remains unknown).^[^ ^148^ ^]^ This has been closely followed by PVA‐based hydrogels using microcrystallization: Here, the PVA/PAAc–Fe^3 +^ double network,^[^ ^99^ ^]^ and the dual cross‐linked PVA–Cit^3 −^ single network^[^ ^90^ ^]^ reach exceptional fracture energy values, while their elastic moduli remain below *E* = 10 MPa. As opposed to the composite materials, however, their stretchability is much improved (ε>1000vs.<350%), additionally^[^ ^90^ ^]^ has the highest water content of this data assembly (90%).

In the range of fracture energies between $\Gamma =20-40\;\mathrm{kJ}\;m^{-2}$, we find double networks of PVA/Alg–Ca^2 +^,^[^ ^97^ ^]^ gelatin/gellan gum–Na^+^,^[^ ^152^ ^]^ followed by the single networks of P(MAAm‐*stat*‐MAAc),^[^ ^71^ ^]^ and P(AAm‐*stat*‐VIm)–Cu^2 +^.^[^ ^51^ ^]^ It should be mentioned that the hydrogen bond generated hydrogels of^[^ ^71^ ^]^ reached an elastic modulus of *E* = 217 MPa, which is one of the highest reported values for tough hydrogels to date. In the range of $\Gamma =10-20\;\mathrm{kJ}\;m^{-2}$, almost all classes of interactions are represented (Figure 10). Together, these data span a range of stiffnesses up to *E* = 100 MPa. The exact value of *E* = 100 MPa is solely achieved through the hydrogen bonding P(NASC) gels.^[^ ^78^ ^]^ This stiffness is followed by the PAAm/CS–polypyrrole double network (*E* = 50 MPa),^[^ ^63^ ^]^ and a dual cross‐linked P(AN‐*stat*‐AAm) single network (*E* = 16 MPa).^[^ ^117^ ^]^ In the range of *E* = 1 − 10 MPa with ascending modulus, we find PAAm/CS–Cit^3 −^ and PAAm/κ‐Carr–Zr^4 +^ double networks,^[^ ^45^, ^56^ ^]^ single networks of PVA/SiO_2_ nanocomposites,^[^ ^125^ ^]^ PDMAAm/lignin,^[^ ^120^ ^]^ a PVA/PAAm double network,^[^ ^95^ ^]^ an ionic PDMAEA‐Q/PNaSS single network,^[^ ^59^ ^]^ and lastly a PAAm/P(SA‐*stat*‐MAAc) SIPN.^[^ ^106^ ^]^

Overall, the use of double networks may seem favorable to reach mechanical properties for load‐bearing applications and indeed, some of the best‐performing specimen are double networks. This however, must not be generalized as several single networks are able to reach extreme mechanical properties as well. Additionally, it should be noted that the upper range of mechanical properties (fracture energy and stiffness) are almost exclusively reserved for tough hydrogels, whose water content is below 50%, with few exceptions, such as.^[^ ^90^ ^]^ This may limit their opportunities in applications, where a high water content is needed.

## 5 Current Applications of Tough Hydrogels

**Figure 11:**
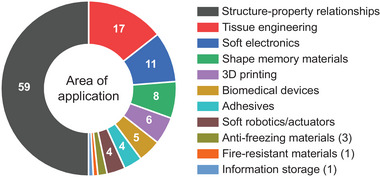
Number of publications related to tough hydrogels cited within this review according to their respective areas of applications.

In this section, we will discuss current targeted applications of tough hydrogels. **Figure** **11** gives an overview of the articles referenced and discussed in the previous section and the respective applications of the presented tough hydrogels. To understand the interactions in the tough hydrogel that renders them suitable for load‐bearing applications, structure–property relationships are at the core focus of investigation of 50% of all cited publications here. This includes changes in the hydrogel precursor formulation (typically concentration of monomer/polymer, cross‐linker, nanoparticles, etc.) and/or variation of process parameters, such as temperature, immersion time, and others.

As a logical extension, many materials are studied and characterized with regard to their self‐healing and ‐recovering properties. As outlined in the first section on tough hydrogel mechanics, this is done by conducting cyclic tensile tests (Figure 4). The system with the best damping capacity is represented by the PAAm/CS–Cit^3 −^ double network, where a total energy of *W*_*hys*_ = 9 MJ m^−3^ was dissipated upon a single tensile cycle (ε=350%).^[^ ^56^ ^]^ In contrast, the system with the lowest hysteresis energy and quasi‐elastic behavior was reported by Lei et al. with their self‐assembling protein‐based hydrogels.^[^ ^156^ ^]^ The latter showed negligeable hysteresis, despite being stretched to a sizable strain of ε=1000% (much like Figure 4b).

When cyclic tensile tests were performed on tough hydrogels, the hysteresis recovery spanned 10–90% (along with tested strains in the range of ε=20−1000%), which makes it difficult to compare individual data sets. Furthermore, the majority of the reported data lied in a much smaller range of $h_{r}=20-40\%$, which reveals the current limitations of self‐healing and ‐recovery independent of the material's improved (fracture) mechanical properties. One notable system with excellent hysteresis recovery was the zwitterionic hydrogel based on sulfobetaine and DMAEA‐Q as reported by Gong and co‐workers.^[^ ^61^ ^]^ Here the hysteresis recovery reached $h_{r}=93\%$ at a maximum strain of ε=350% (much like Figure 4d).

Reduced recovery ratios arise due to irreversible changes in the network during the energy dissipation (e.g., breaking bonds). In certain cases, however, the mechanical history of the sample can be erased by giving it time to rest and relax. For a swift recovery from a potentially cyclical impact, materials that require shorter resting times are preferred. Most commonly, tough hydrogels were given 5–10 min to relax between two tensile cycle. The hydrogen bonding networks based on P(VIm‐*stat*‐MAAc) exhibited one of the most efficient self‐recovering behavior.^[^ ^74^ ^]^ In the related report, the gel specimen was elongated to ε=300% before being given 10 min to relax at room temperature. Upon a second tensile cycle, the hysteresis recovery reached $h_{r}=91\%$. The single network made from partially hydrolyzed carboxy methyl chitosan and PAAm reached higher values: after a 2 min recovery period, the hysteresis recovery was $h_{r}=100\%$ when stretched to ε=500%.^[^ ^65^ ^]^ The last example of an extremely fast self‐recovering gel was represented by the micellar PAAm/DVB hydrogels, where relaxing the hydrogel 3 min at room temperature was sufficient to give a hysteresis recovery of $h_{r}=100\%$ at a strain of ε=700%.^[^ ^111^ ^]^ To fully recover ($h_{r}=100\%$), other tough hydrogels required longer relaxation times,^[^ ^54^, ^58^, ^59^, ^70^, ^83^, ^103^ ^]^ and/or elevated temperatures.^[^ ^44^, ^112^ ^]^

Aside from the above works focusing on the fundamentals, tissue engineering remains the most popular area of application for tough hydrogels. This mostly involves testing the gel's in vitro cytocompatibility,^[^ ^42^, ^72^, ^94^, ^128^, ^145^ ^]^ in vivo injectability or capacity to act as implants.^[^ ^63^, ^82^, ^98^, ^141^ ^]^ Two reports envisaged tough hydrogels as skin replacements and have indeed tested their wound healing capacities.^[^ ^63^, ^141^ ^]^ For comparative reasons, the papers by Wegst and Ashby, as well as Taylor and co‐workers are widely cited for the fracture mechanics of soft biological tissues.^[^ ^178^, ^179^ ^]^ Wegst and Ashby, however, reported data based on gekko lizard skin ($\Gamma =0.5-2\;\mathrm{kJ}\;m^{-2}$),^[^ ^180^ ^]^ rather than human skin, which was shown to have a fracture energy of $\Gamma =3.6\;\mathrm{kJ}\;m^{-2}$ (stratum corneum).^[^ ^181^ ^]^ In this regard, the earlier cited work involving the PAAm/CS–polypyrrole gels ($\Gamma =12\;\mathrm{kJ}\;m^{-2}$, *E* = 50.1 MPa) reached much higher values than needed.^[^ ^63^ ^]^ The article by Zhang et al.,^[^ ^141^ ^]^ which also looked into skin wound healing, was in good literature agreement regarding the fracture energy ($\Gamma =3.25\;\mathrm{kJ}\;m^{-2}$), yet their tensile modulus (*E* = 7 kPa) did not match measured values of, for instance, tibial skin grafts (*E* = 0.3 − 20 MPa).^[^ ^182^ ^]^ Additionally, the mechanical properties of skin often vary with skin type, thickness, layer, age and orientation. Consequently, these two cases highlight the complexity of matching synthetic material properties to human tissue and the discrepancies that often arise between them.

**Figure 12:**
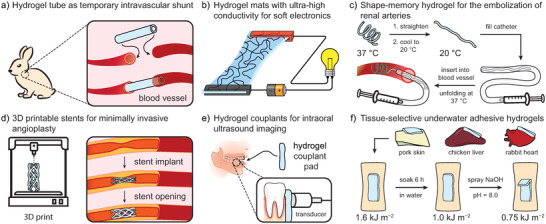
Chosen examples of tough hydrogels applied in the areas of a) tissue engineering, b) soft electronics, c) shape memory materials, d) 3D printing, e) biomedical devices, and f) adhesives. Notably, the majority of the highlighted examples ultimately serve biomedical applications.

Similar trends are perceived when it comes to implantable gels, which are often tested as drug delivery vehicles and have shown to promote anti‐bacterial, anti‐inflammatory, or anti‐fouling properties.^[^ ^50^, ^78^ ^]^ One such example effectively created a hydrogel tube (PNASC) that was tested as an artificial blood vessel in rabbits (**Figure** **12**a). The authors implanted a 3 cm long hydrogel tube and did not observe hemorrhage after the implantation (vascular anastomosis) for 4 h.^[^ ^78^ ^]^ Among the few types of blood vessels studied, the fracture energy of pig aorta was $\Gamma =1.8\;\mathrm{kJ}\;m^{-2}$.^[^ ^183^ ^]^ Whether this value is similar to rabbit or human tissue, however, is still unknown.

Owing to their enhanced fracture mechanics, many materials target the replacement of tough tissues, such as cartilage, muscle (ligaments, tendons), or even bone. Cartilage tissue is often described to have a fracture energy of $\Gamma =0.2-1.2\;\mathrm{kJ}\;m^{-2}$, although these values are measured for canine cartilage.^[^ ^184^ ^]^ Analogous to skin, it is well known that cartilage fracture properties can vary significantly between left or right cartilage,^[^ ^185^ ^]^ depending on the collagen fiber orientation and tissue location,^[^ ^186^ ^]^ and within different animal specimen and across species.^[^ ^184^ ^]^ Hence, human articular cartilage may differ from what has been reported for animals. Overall, this analysis reveals a substantial knowledge gap regarding the fracture mechanics of human tissue and more studies on the fracture mechanics of biological tissues are needed.

The area of second most popularity is soft electronics and tough hydrogels have predominantly been used as strain sensors, where a deformation of the material leads to a measurable change in electrical properties. Consequently, their characterization most commonly includes conductivity measurements (often with varying strain of the sample), spanning a conductivity range of 0.3–1.66 · 10^4^ S m^−1^. A conductivity of 1.66 · 10^4^ S m^−1^ was the highest reported value for conductive tough hydrogels and was achieved in a PVA‐based composite gel embedded with polyaramid fibers and Ag nanowires (Figure 12b).^[^ ^146^ ^]^ Due to the high conductivity, the materials exhibited high shielding capabilities from electromagnetic interference, which is especially desired in soft robotic applications to avert electric malfunctions. As opposed to other literature‐reported strain sensors, their conductivity stayed constant for 500 cycles over a strain range of ε=10−90%, with the specific need for this strain‐independence remaining somewhat unspecified.

Tough hydrogels with shape memory properties have the ability to remember their original shape and return to it via an external trigger once they have been deformed. Such materials are of interest for biomedical applications, where they can be implanted in a less invasive deformed state and once put in place are triggered to return to their original shape, performing an intended function. Zhang et al. demonstrated this with their P(AN‐*stat*‐AAm) single networks (driven by hydrophobic interactions), which were used for the embolization of porcine renal arteries to counteract aneurysm‐caused hemorrhages.^[^ ^117^ ^]^ The hydrogels were injected via a catheter in an unfolded state and once they reached a temperature of 37°C coiled up tightly to block the respective artery (Figure 12c). Angiographic images showed that the embolization was successful and stable for up to 12 weeks; no recanalization was observed and when removed the hydrogel string was still tightly entangled. The fracture energy of this material ($\Gamma =16.6\;\mathrm{kJ}\;m^{-2}$), however, was tenfold that of pig aorta and how this value benefited the hydrogel string was not discussed. Nonetheless, the above example shows nicely how tough hydrogels can be used to replace contemporary devices that are often based on metal alloys.

The 3D printing of hydrogels has various advantages, such as design flexibility, rapid prototyping, realization of complex structures, while at the same time it reduces material waste. Most 3D printed soft hydrogels find application as drug delivery systems or in tissue engineering, yet, to form stiff and tough hydrogels through printing of liquid precursor solutions can still be a challenging endeavor. Wu et al. used directed light processing to print stents based on their P(NASC‐*stat*‐AAc) polymer networks (Figure 12d).^[^ ^79^ ^]^ The formulation they used in this approach reached outstanding mechanical properties of *E* = 7 MPa and $\Gamma =75\;\mathrm{kJ}\;m^{-2}$, which exceeded many of their non‐printable competitors. The hydrogen bonding of the tough hydrogels was temperature‐responsive: at 60°C the urea–carboxyl interactions were broken. For a minimally invasive angioplasty, the hydrogel can be first compressed at 60°C, then fixated at 5°C, ready to be implanted into the blood vessel. In a cardiovascular simulation of blood vessel stenosis, the cytocompatible stent was shown to expand into its original shape and stayed in place at 37 °C. In vivo experiments, however, have not yet been realized and would represent a valuable continuation of this work.

Apart from 3D printing, the direct fabrication of biomedical devices based on tough hydrogels has been the subject of multiple studies. This involves the creation of a biomedical tool based on tough hydrogel architectures that is not used for tissue engineering but to facilitate operation or post‐operative care. The previous examples on vascular embolization and stents demonstrate this well,^[^ ^79^, ^117^ ^]^ and other reports have used tough hydrogels as wound sutures,^[^ ^99^ ^]^ tubular graspers,^[^ ^52^ ^]^ or for intraoral ultrasound imaging.^[^ ^38^ ^]^ To improve intraoral ultrasound imaging of dento‐periodontal tissues, PAAm/Alg–Ca^2 +^ double networks were used as couplant gel pads between transducer and oral tissue (Figure 12e). The enhanced mechanical properties of the tough hydrogels make them ideal targets to replace current commercial water‐unstable, and rather brittle gel pads in an attempt to rival conventional x‐ray examinations.

Adhesive hydrogels are relevant for a variety of applications, such as in implants, tissue repair, for underwater sensoring, or marine repair works. To this end, it is essential to provide the necessary chemical properties to the hydrogel for it to be able to adhere to the desired surface. Liu and co‐workers demonstrated this by combining alkylcatechol‐containing copolymers with CS to obtain a tough double network hydrogel.^[^ ^64^ ^]^ The catechol unit promoted selective and improved adhesion toward wet biological tissue, as well as CS or gelatin surfaces, rather than plastic, metal, or rubber (Figure 12f). Since the fracture energy of the evaluated hydrogel ($\Gamma =6.6\;\mathrm{kJ}\;m^{-2}$) lied in the same range as the one of porcine skin ($\Gamma =1.6\;\mathrm{kJ}\;m^{-2}$), the latter was primarily used to quantify the hydrogel's adhesiveness in a lap shear adhesion test. The hydrogel showed an adhesion strength of 180 kPa for 20 cycles, an adhesion energy of 1.6 kJ m^−2^, which decreased to 1 kJ m^−2^, when the porcine skin‐hydrogel composite was immersed for 6 h in water. With an aqueous solution (pH = 8), the hydrogel's adhesion energy was further weakened to facilitate dettachment of the hydrogel from the porcine skin leaving it damage‐free. These results showed how the fracture and adhesion energy can ben used as quantitative tools, their change either allowing the gel removal or promoting strong surface bonding.

A smaller fraction of tough hydrogels has been applied as soft actuators,^[^ ^40^, ^51^, ^137^, ^147^ ^]^ as anti‐freezing gels,^[^ ^36^, ^62^ ^]^ fire‐resisting materials,^[^ ^105^ ^]^ as well as in information storage.^[^ ^129^ ^]^ Cui et al. reported a micellization‐driven double network hydrogel based on Alg–Li^+^/P(AAm‐*stat*‐SMA)–SDS. The authors argued that the ideal fire‐resistant hydrogel required mechanical toughness, self‐healing and water‐retaining properties and hence, made a case study with their materials. When the LiCl content in the hydrogels was increased (>4.0 m), the ratio of ion‐bound water to free water increased, leading to enhanced water retaining properties of the hydrogels. This came, however, at the expense of the self‐healing behavior, which passed a maximum of 35% at 1.0 m. Altogether, a 2 mm thick hydrogel was capable of resisting a high temperature flame spray for 45 s.

To recapitulate, we encountered many different application areas for tough hydrogels, yet only selected examples achieved high impact through combination of careful experimental design and material analysis. The majority of studies in this review focus on the hydrogel's structure–property relationship, self‐healing, and self‐recovery behavior and as a logical continuation, the exploration of specific, targeted applications would be desirable. Among the applied studies, biomedical applications are by far the most popular choice, which include the direct use of gels as tissue engineering scaffolds (e.g., wound healing), as well as the fabrication of biomedical devices for (post‐)operational use. Besides, opportunities of tough hydrogels in soft electronics, actuators or 3D printing are increasingly explored, which often however, represent just an intermediary step toward their final application in the biomedical field. Some reports ensure good correlation between mechanical properties of the gels and the tissue beforehand, while in other instances the assumption prevails that deviating or exceeding properties will still satisfy the needs of the targeted (tissue) application. This being said, matching the material's properties to the target environment is less prevalent in soft electronics or actuators, where the need for specific (fracture) mechanical properties are often not further explained. Nonetheless, a broad range of (fracture) mechanical properties has become accessible. Consequently, the question arises, whether this may open up new areas of applications, which will be subject of the final and last section of this review.

## 6 Future Perspectives

**Figure 13:**
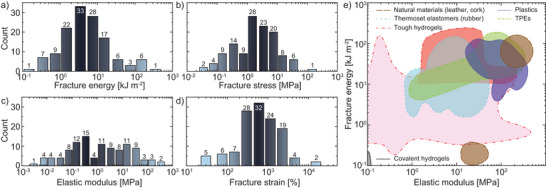
Statistic distributions on tough hydrogels properties of the analyzed literature for fracture energy (a), elastic modulus (c), fracture stress (b), and fracture strain (d). e) Ashby plot of chosen natural (brown) and synthetic materials (plastics: blue, thermosets/rubbers: turquoise, TPE: green) together with covalent (grey) and tough hydrogels (pink). Tough hydrogels with most extreme properties from Figure 10 are shaded red. The depicted data is limited exclusively to values in between $\Gamma =0.1-1000\;\mathrm{kJ}\;m^{-2}$ and *E* = 0.1–1000 and was partially sourced from Ansys GRANTA EduPack software © (the graphic was recreated).

The range of mechanical properties currently available by the new generation of tough hydrogels is vast. In this section, we will relate said mechanical properties with those of commercial materials. This will help to give a clearer perspective of current tough hydrogels and their potential to be engineered for load‐bearing applications. In a statistical sense, the majority of tough hydrogels has a fracture energy in between $\Gamma =2-5\;\mathrm{kJ}\;m^{-2}$, followed by $\Gamma =5-10\;\mathrm{kJ}\;m^{-2}$ and $\Gamma =10-20\;\mathrm{kJ}\;m^{-2}$ (**Figure** **13**a). Fracture energies exceeding $\Gamma =20\;\mathrm{kJ}\;m^{-2}$ are rare, but have recently been reported as seen in the main section of this review. The wealth of elastic moduli among the tough hydrogels is distributed over a much broader range, six orders of magnitude (Figure 13c). Regarding the elastic moduli, a much broader distribution is observed with only a small fraction of hydrogels exceeding *E* = 50 MPa.

Real world materials in this specific range of properties comprise some natural materials (leather or cork), thermoplastics, thermoplastic elastomers (TPE) and thermosets or rubbers (Figure 13e). Thermoplastics with elastic moduli higher than 50 MPa predominantly comprise poly(vinyl chloride) (PVC) and various ionomers (polymers with a share of ionic groups in the backbone). A classic use of PVC is cable sheathing, where a stiff and fracture‐resistant shell or coating is needed to protect the underlying cable from being exposed to potentially harsh or damaging environments. The stretchability of soft PVC is mostly limited to $\varepsilon_{b}=300\%$, which is where tough hydrogels could pose a distinct advantage as their failure strains often exceeds $\varepsilon_{b}=500\%$ (Figure 13d). This could be of interest for coating technologies in marine conditions or those, where the presence of aggressive solvents or oil would render ordinary coatings brittle due to the gradual removal of plasticizers.

Ionomers can be engineered to have elastic moduli of *E* > 50 MPa and in the presence of specific ions become even tougher due to enhanced interactions between the charged polymer chains and the ions. This has mostly been exploited for coatings and could be translated to tough hydrogels based on metal–ligand interactions. Moreover, ionomers have been used for semi‐permeable membranes (e.g., in batteries) and tough hydrogels could be applied in a similar manner toward emerging aqueous‐based battery technologies (e.g., aqueous redox flow batteries).

The thermoplastic elastomers in Figure 13e include copolyesters, copolystyrenes, polyurethanes, and others. They often are blends between small amounts of thermoplast and fractions of rubber elastomer and can be extruded. The stiffer ones are mainly based on thermoplastic polyurethane and are primarily used in biomedical applications, for instance, as tubing. The remaining share of TPEs with *E* < 50 MPa exhibit elastic moduli similar to traditional elastomers, yet the latter span a larger range of fracture energies $\Gamma =1-100\;\mathrm{kJ}\;m^{-2}$. As such, the portrayed elastomers include natural rubbers (unfilled and filled), perfluoro, nitrile, polysulfide, silicon elastomers, among others. Elastomers have been applied in a myriad of components, such as rubber seals, gaskets, cable sheathing, (cable) insulation, O‐rings, complex molds, gloves, car tires, hoses, etc. Most elastomers are hydrophobic, which makes them resistant to water, however, they can be prone to swelling with organic solvents, oils or other petrol‐based contaminants. This could open up new opportunities for tough water‐born hydrogels, which can reach similar elasticities, elastic moduli, and fracture energies, yet may be more resistant to organic contaminants on account of their hydrophilic nature. In environments where traditional elastomer parts have to be replaced on a periodical basis, such hydrophilic tough and elastic hydrogels could be employed for long‐term usage if their water content can be retained over time. In an ideal case, such hydrogel‐based seals would reject solvents and therefore behave like inert and isolating materials. In line with this, such tough hydrogel coatings could be of interest in environments where it is desired to keep moisture around and contain (high) amounts of water. This could be of interest as coatings for moisture regulating surfaces in construction but also in closed environments, such as storage tanks or containers.

## 7 Closing Remarks

The soft matter hydrogel community has come a long way in the development of hydrogel architectures that satisfy a broad range of properties and applications. With the emergence of tough hydrogels for load‐bearing applications, new territories were charted, especially in the (bio)medical area. Many systems are increasingly accessible, for instance, the likes that make use of polysaccharides, PVA, and ionic or hydrogen‐bonding monomers. For the majority of materials, free‐radical polymerization has been the method of choice due to its ease of use and tolerance to various solvents, monomers, pH values, temperatures and manufacturing conditions. The increased availability combined with the simplicity of the building blocks and methods will progressively reduce production costs, and render the resulting materials more attractive and competitive toward today's established high‐performance materials.

Throughout this review, we have seen that innovative cross‐linking strategies often give rise to a broad span of mechanical properties, producing—for the first time—fracture energies and elastic moduli that exceed $\Gamma =100\;\mathrm{kJ}\;m^{-2}$ and *E* = 100 MPa, respectively (this was enabled specifically through PVA microcrystallization, hydrogen bonding and composite materials). Metal–ligand interactions with biopolymers using alkaline earth and alkali metals have become popular due to their biocompatibility that enables easier implementation into biomedical applications. They are frequently combined with covalent networks to give double networks that manage to reconciliate good fracture energies ($\Gamma <20\;\mathrm{kJ}\;m^{-2}$) with high stretchabilities (>1000%); their stiffness, however, remained limited (*E* < 2 MPa). Transition metal–ligand interactions have shown to effectively improve stiffness (*E* = 10 − 20 MPa), however, both their water content and biocompatibility is generally lower, especially when it comes to higher cross‐linker concentrations. A similar trend is observed for tough hydrogels based on ionic interactions, where a sufficiently high concentration is needed to enable gelation along with enhanced (fracture) mechanical properties ($\Gamma <20\;\mathrm{kJ}\;m^{-2}$). Due to their ionic nature, such hydrogels are often applied as conductive strain sensors and more generally in soft electronics. In the category of hydrogen bonding, a wide range of stiffness and fracture energies (*E* < 220 MPa, $\Gamma <144\;\mathrm{kJ}\;m^{-2}$) is observed, which is mainly driven by the introduction of new innovative monomers capable of strongly interacting with each other. The higher stiffness values require higher monomer concentrations and thus, come at the expense of the water content (lowered to 40–60%). Nonetheless, the creative flexibility in monomer design, their benign chemical nature and ease of production has brought forward some of the most promising tough hydrogels with biomedical application.

For microcrystallization, PVA remains the primary resource when it comes to facile production of tough hydrogels, as it is a widely available and biocompatible polymer. Additionally, more advanced curing techniques such as directed freeze‐casting or combinations of freeze‐thawing with salting‐out, metal–ligand, or ionic interactions have pushed boundaries of fracture mechanical properties ($\Gamma >100\;\mathrm{kJ}\;m^{-2}$). By contrast, such hydrogels often suffer from more complicated fabrication procedures and higher salt/metal concentrations often entail lower water contents. Tough hydrogels obtained through micellization combine good fracture energies ($\Gamma <40\;\mathrm{kJ}\;m^{-2}$) with Young's moduli mostly in the kPa range and largely varying water contents. Further to this, formulations often comprise complex component mixtures (different monomers/polymers, surfactants, etc.) to drive the hydrophobic self‐assembly processes and obtain desired material properties. Microphase separation via hydrophobic interactions exhibit similar limitations, however, in average they produce higher stiffness (as shown by hydrogel examples using acrylonitrile, *E* < 145 MPa). They are, however, often fabricated in organic media prior to a solvent exchange to water; this puts them at a disadvantage when compared to materials directly obtainable from aqueous solutions. Nonetheless, tough hydrogels based on micellization or hydrophobic interactions have covered a broad application range, including skin‐inspired wearable sensors, conductive soft electronics, 3D printing, fire‐resistant materials and various biomedical applications.

Composite materials have set new heights in both stiffness and fracture energies (*E* < 420 MPa, $\Gamma <207\;\mathrm{kJ}\;m^{-2}$). Among tough hydrogel composites, the most popular additives are silica NPs or clay nanosheets, which are increasingly commercially available and can be easily functionalized and incorporated into networks using established protocols. Especially for the stiffest samples, however, stretchability, as well as water content suffer greatly due to the added solid content. This is especially the case for systems that make use of in situ mineralization processes or embedded (nano)fibers (ε<100%). Regarding their applications, nanocomposite hydrogels that use mineralization processes predominantly target osteochondral repair, mimicking the CaP generation in the bone structure. Other systems are applied across all fields in soft electronics, sensoring, tissue engineering, or 3D printing, showcasing their versatility.

The literature examples in the category of self‐assembly are still few in number, yet selected examples using protein folding or host–guest interactions have shown great potential for the production of tough hydrogels. Especially networks with unique topologies (such as slide‐rings) managed to combine outstanding stretchabilities (ε>10000%) with good fracture energies and we believe that, although they are synthetically more challenging, such topologies merit further exploration.

Overall, the discussed literature on tough hydrogels gave key insights into structure–property relationships and illustrated, how individual parameters, such as fracture energy (or fracture strain) often come at the expense of others (e.g., stiffness). The careful adjustment of parameters remains therefore critical to match physicochemical and (fracture) mechanical properties of tough hydrogels to those of a given targeted environment (e.g., various tissues). As witnessed by the presented data charts, the hydrogel's water content is often forgotten but plays an essential role for both the (fracture) mechanical properties and the area of application.

Concerning the mechanical properties, increased water contents generally cause a reduction of stiffness, strength, and fracture energy, while fracture strains can often be enhanced. This effect is largely attributed to the dilution of the network and the neighboring cross‐links, as non‐covalent interactions are increasingly weakened (especially in the case of electrostatic interactions). On the microscale, the molecular distribution of water within a given hydrogel seems to be even more consequential, as could be seen in microphase‐separated systems. Here, water inhomogeneities (e.g., micelles) systematically promoted modes of energy dissipation, resulting in large enhancements of the mechanical properties. Therefore, we believe that water content on the macro‐ and the microscale is often underestimated, its intruiging role for energy‐dissipating polymer networks is often overlooked and merits more attention.

Regarding the area of application, the water content is a vital parameter when considering the biomedical or tissue engineering fields, where within a few mm depth of a single tissue, it may vary vastly (between 20–90%) and tied to it, the (fracture) mechanical properties of the same tissue. Nonetheless, while biomedical applications are often at the very center of attention for many of the highly specialized materials, we should remain receptive to less prominent areas of application including those that reside outside of the traditional hydrogel scope.

This being said, we urge researchers to adhere to comprehensive and detailed characterizations to produce complete datasets, since many reports occasionally miss data on water content or other variables (e.g., elastic modulus). Complete datasets benefit everyone, including the overall landscape of mechanical properties, as well as the general understanding of the underlying interactions and cross‐linking mechanisms within the tough, high‐performance hydrogels. Finally, with this review we hope to have given a detailed overview on the state‐of‐the‐art, cutting‐edge tough hydrogels, unraveled strengths and shortcomings of current systems, and pointed out possible directions and opportunities for prospective applications.

## Conflict of Interest

The authors declare no conflict of interest.

## Supporting information


Supporting Information
