# Supplementary material for: Tough Hydrogels for Load‐Bearing Applications
Source: Adv Sci (Weinh). 2024 Jan 15;11(12):2307404. doi: 10.1002/advs.202307404 (PMC10966577; doi:10.1002/advs.202307404)
Supplement: Supplementary file 1 — Supporting Information [file ADVS-11-2307404-s001.pdf]

ADVANCED SCIENCE
Open Access

Supporting Information

for *Adv. Sci.*, DOI 10.1002/advs.202307404

Tough Hydrogels for Load-Bearing Applications

*Nika Petelinšek and Stefan Mommer**

Supporting Information

Tough Hydrogels for Load-Bearing Applications

Nika Petelinšek[a] and Stefan Mommer[a]*

Table of Contents

1 Methods ... 2

2 Additional plots ... 3

3 Literature data ... 9

[a] *Macromolecular Engineering Laboratory, ETH Zurich, Sonneggstrasse 3, 8092 Zurich, Switzerland.*

* *Corresponding author. E-mail: smommer@ethz.ch*

1 Methods

Literature Survey. Research papers were collected using Clarivate Web of Science. Therefore, certain data included herein are derived from Clarivate *Web of Science*. © Copyright Clarivate 2023. All rights reserved. The published articles were searched according to the search criteria "tough hydrogels" together with "fracture toughness" and/or "fracture energy". This way we made sure that the articles referred to in this review do include fracture mechanics in their analysis of the mechanical properties of tough hydrogels. Since a major focus of this review was to compare the fracture mechanics of tough hydrogels, all the works that lacked this information were deemed beyond this review.

Data collection. The individual values for the mechanical parameters were extracted from each paper and replotted in corresponding benchmark graphs located in the main text and here in the ESI. Usually, research papers produced and analyzed multiple hydrogel samples. Only the gels with the best combined properties were chosen from each article. This choice is often already made by the authors of the publication, many of them choosing a hydrogel specimen among many that is then subjected to additional, more detailed characterization as well as tested for a specific application. In general, we attempted to extract the following data parameters: Components of the Hydrogel, solvent, sample name of the data point, water content, elastic modulus, slope range used for its calculation, sample dimensions for tensile testing, gauge length L_0, strain rate, tensile strength (fracture stress), elongation at break (fracture strain), toughness (W), fracture energy (Γ), notch length, fracture testing method (PS for pure shear, Tr for Trouser tearing test), sample dimensions for fracture testing, strain rate for fracture testing, standard norm followed for the test (not always specified), hysteresis energy and its maximum elongation. For cyclic tensile tests often referred to as fatigue resistance we describe which mode is being used (T for tensile test, C for compressive test). Related data includes strain rate, hysteresis recovery. For self-recovery properties we collected data on the hysteresis recovery after a recovery time (within 5-10 minutes) and the maximum achieved hysteresis recovery (h_{max}) after a recovery time x, as well as the maximum strain the sample was subjected to and the temperature of the experiment.

Data extraction. Whenever possible, data was collected from text and tables in the main manuscripts and/or supporting information. In many cases, only limited amount of data was written in numbers and while graphics often have shown the complete datasets, they are only visual representations of values that were needed for this review. In these cases, we made use of im2graph (version 1.21, https://www.im2graph.co.il/). Im2graph is a free software, that can convert graphs and plots into data. The boundaries of X- and Y-axis serve as input for the algorithm to allow read-out of selected datapoints on a plot. This way we extracted data that was not written down in text. All data that was manually read out by use of im2graph is marked in orange as it represents no absolute values but read-out values. Further, the data for water content was often not given and therefore calculated by us. This was done by

following the experimental procedure, where we calculated the sum of all solids used in a given protocol and divided it by the sum of the solid contents and total mass of solvent used. This calculation was only done in those cases, where the as-prepared hydrogels were used directly for mechanical testing without further swelling, soaking or immersing in other liquids. For hydrogel samples that were immersed into salt or aqueous solutions as part of the preparation or post-processing, the water content was not calculated, since such immersion processes often results in swelling or deswelling of the hydrogel specimen making a calculation based on the preparation protocol redundant. Again, all calculated values for water content or those that have been extracted from plots via im2graph are labelled as orange text and therefore are no absolute values but rather calculated or read-out values.

Referencing. The numbering of the citations in this document is aligned with the numbering of the citations in the main manuscript. This way, supporting information in this document can be easily read out together with the main text of the manuscript and is tied to a fixed number, which consequently and in the case of this document does not begin with number 1.

2 Additional plots

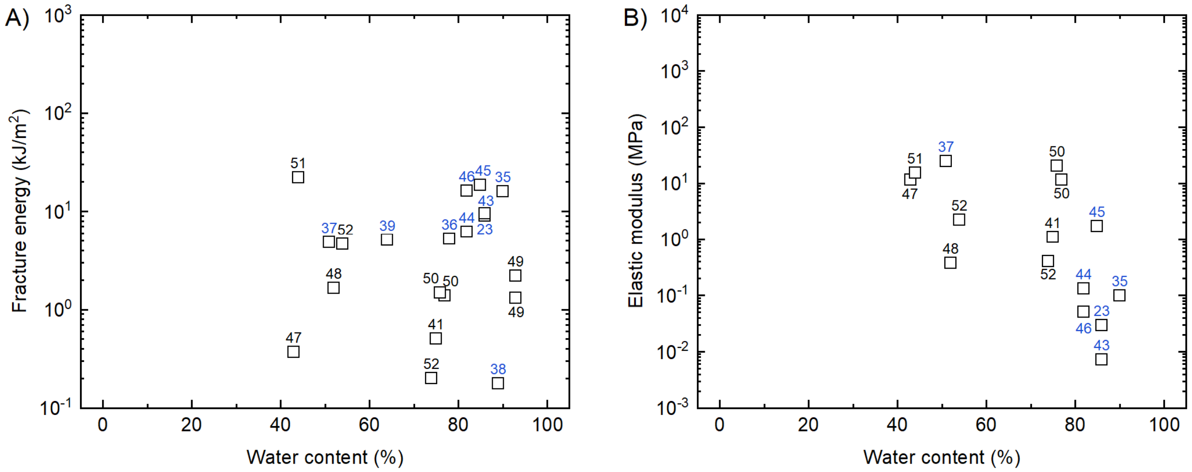

Figure S1. A) Fracture energy and B) elastic modulus vs. water content for tough hydrogels using *metal ligand interactions*. Labels refer to citations found in Table S1 (SN: black; DN: blue).

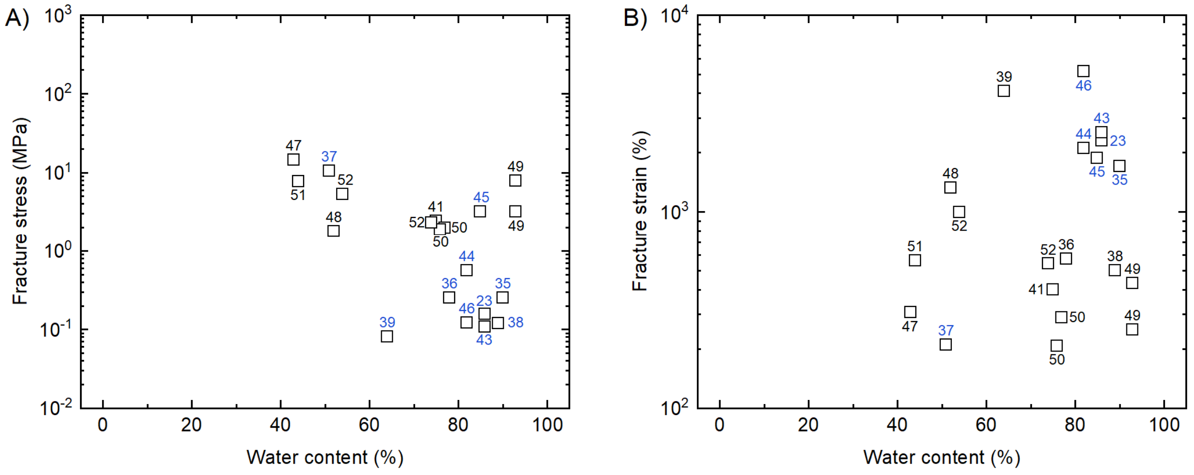

Figure S2. A) Fracture stress and B) fracture strain vs. water content for tough hydrogels using *metal ligand interactions*. Labels refer to citations found in Table S1 (SN: black; DN: blue).

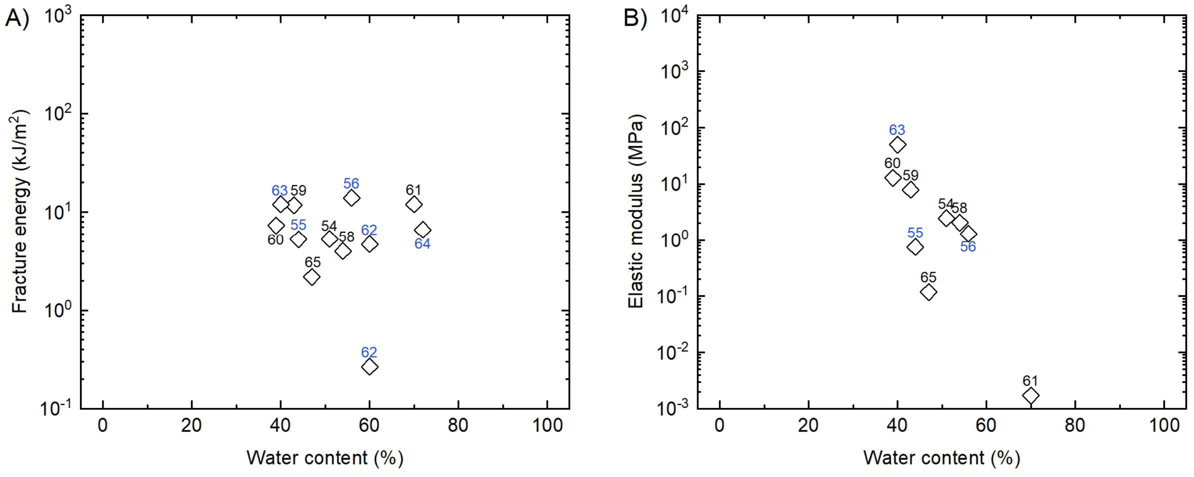

Figure S3. A) Fracture energy and B) elastic modulus vs. water content for tough hydrogels using *ionic interactions*. Labels refer to citations found in Table S1 (SN: black; DN: blue).

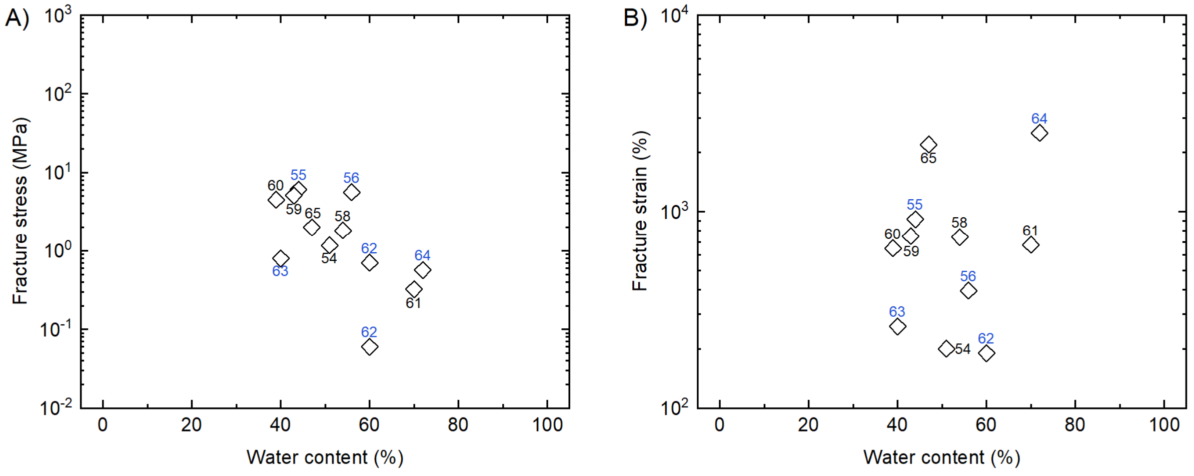

Figure S4. A) Fracture stress and B) fracture strain vs. water content for tough hydrogels using *ionic interactions*. Labels refer to citations found in Table S1 (SN: black; DN: blue).

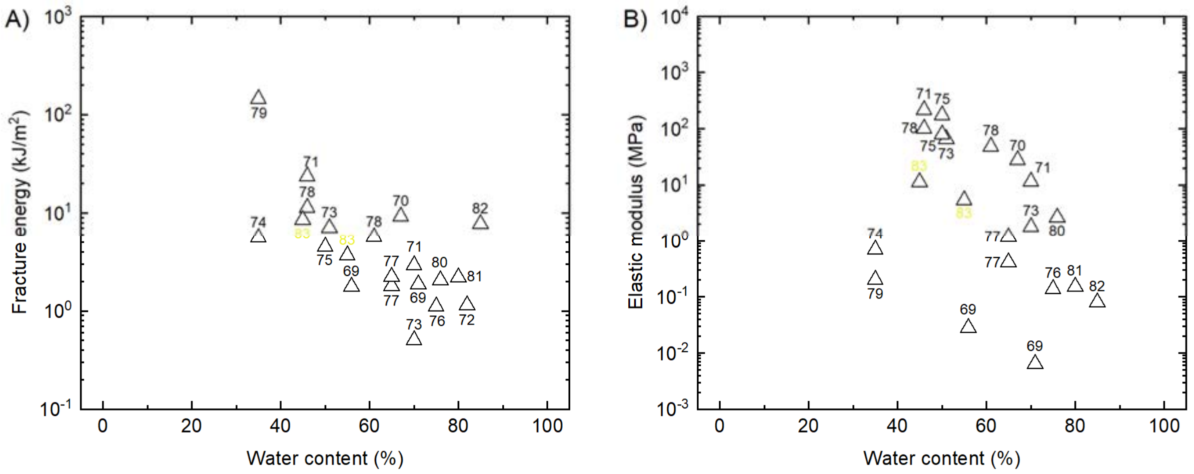

Figure S5. A) Fracture energy and B) elastic modulus vs. water content for tough hydrogels using *hydrogen bonding interactions*. Labels refer to citations found in Table S1 (SN: black; SIPN: gold).

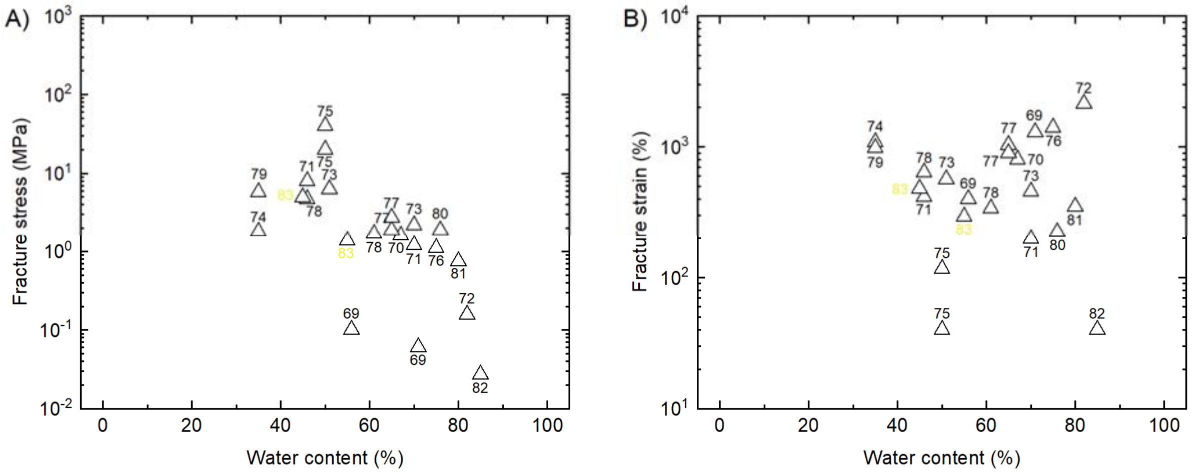

Figure S6. A) Fracture stress and B) fracture strain vs. water content for tough hydrogels using *hydrogen bonding interactions*. Labels refer to citations found in Table S1 (SN: black; SIPN: gold).

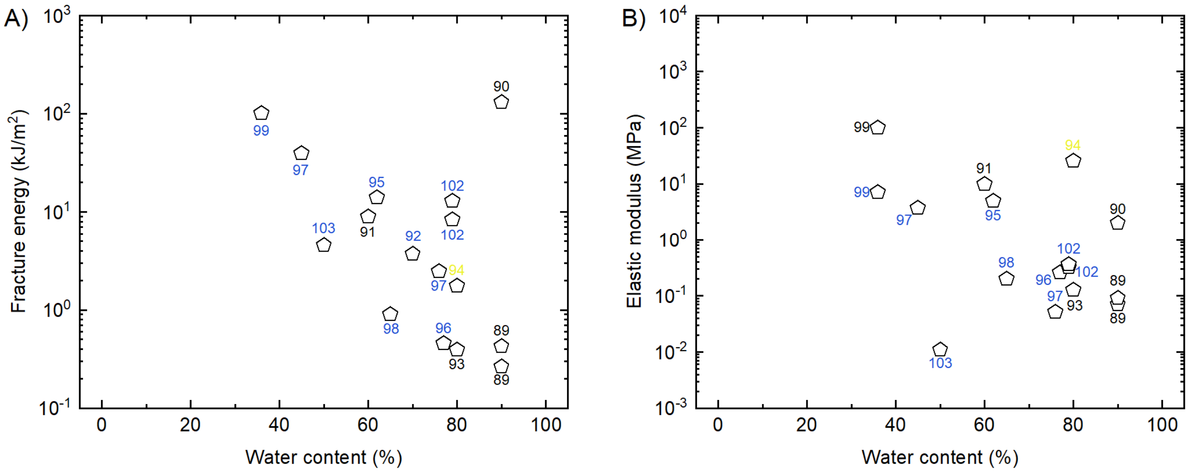

Figure S7. A) Fracture energy and B) elastic modulus vs. water content for tough hydrogels through *crystallization/salting out*. Labels refer to citations found in Table S1 (SN: black; SIPN: gold; DN: blue).

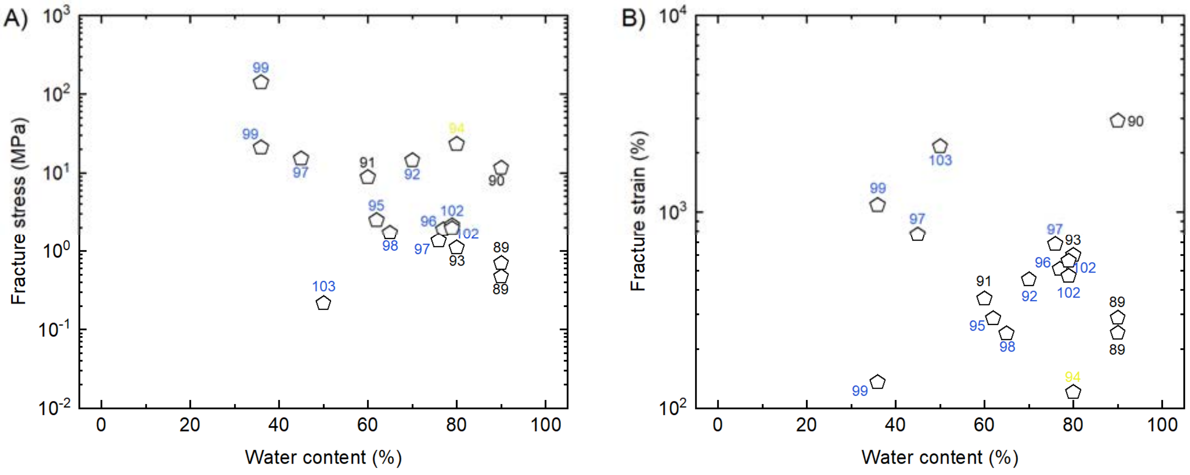

Figure S8. A) Fracture stress and B) fracture strain vs. water content for tough hydrogels through *crystallization/salting out*. Labels refer to citations found in Table S1 (SN: black; SIPN: gold; DN: blue).

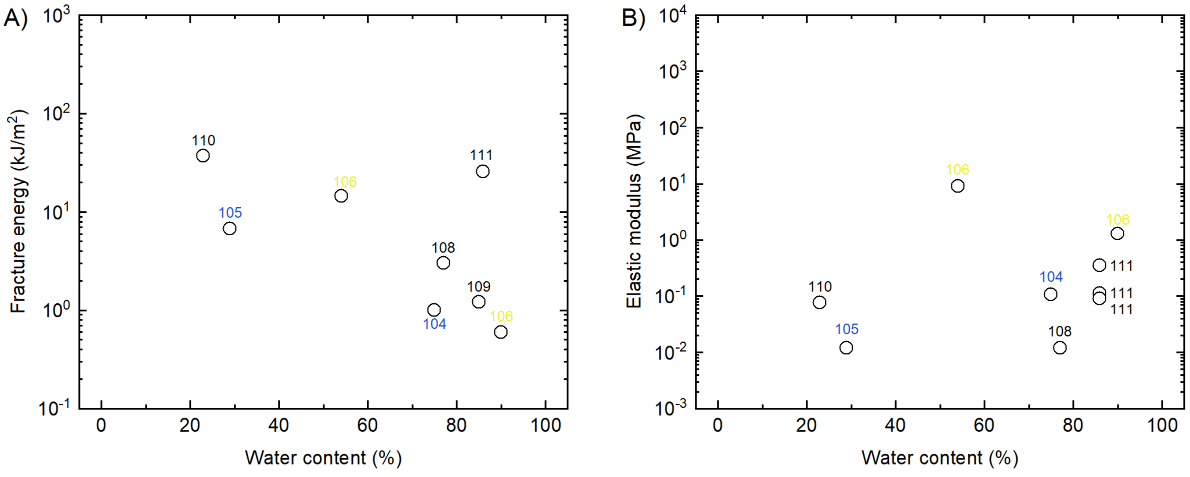

Figure S9. A) Fracture energy and B) elastic modulus vs. water content for tough hydrogels through *micellization*. Labels refer to citations found in Table S1 (SN: black; SIPN: gold; DN: blue).

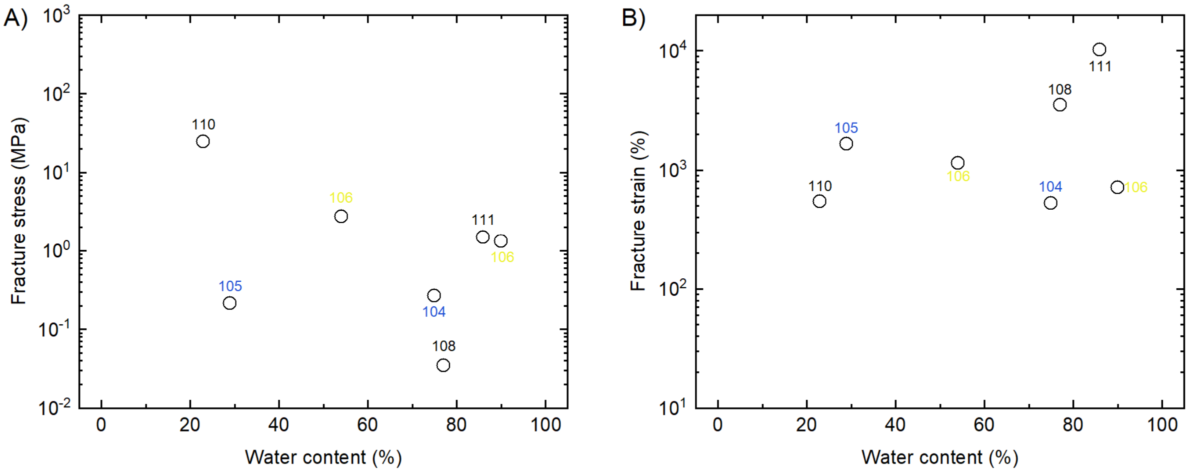

Figure S10. A) Fracture stress and B) fracture strain vs. water content for tough hydrogels through *micellization*. Labels refer to citations found in Table S1 (SN: black; SIPN: gold; DN: blue).

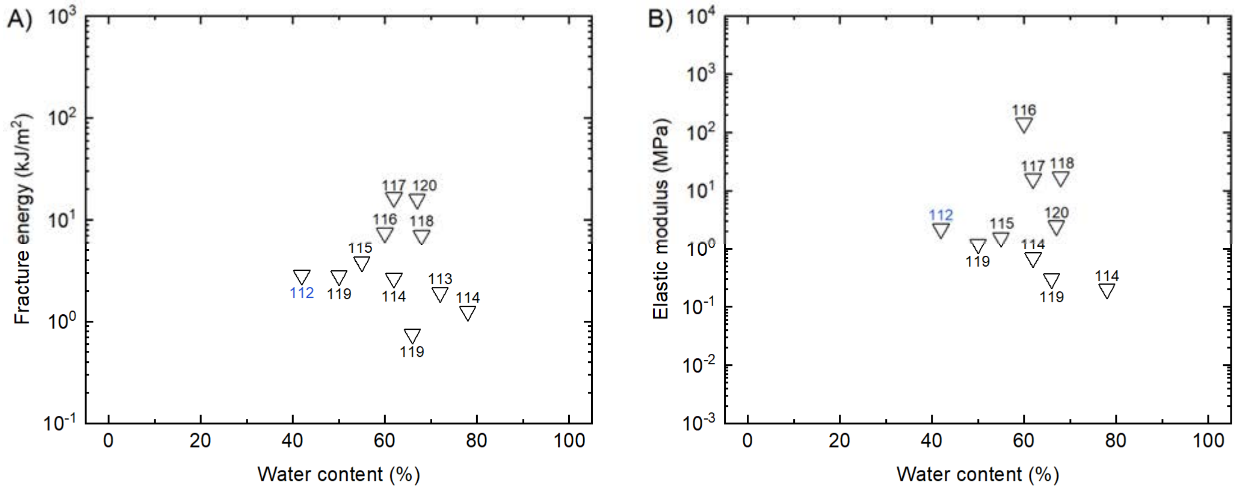

Figure S11. A) Fracture energy and B) elastic modulus vs. water content for tough hydrogels through *hydrophobic interactions*. Labels refer to citations found in Table S1 (SN: black; DN: blue).

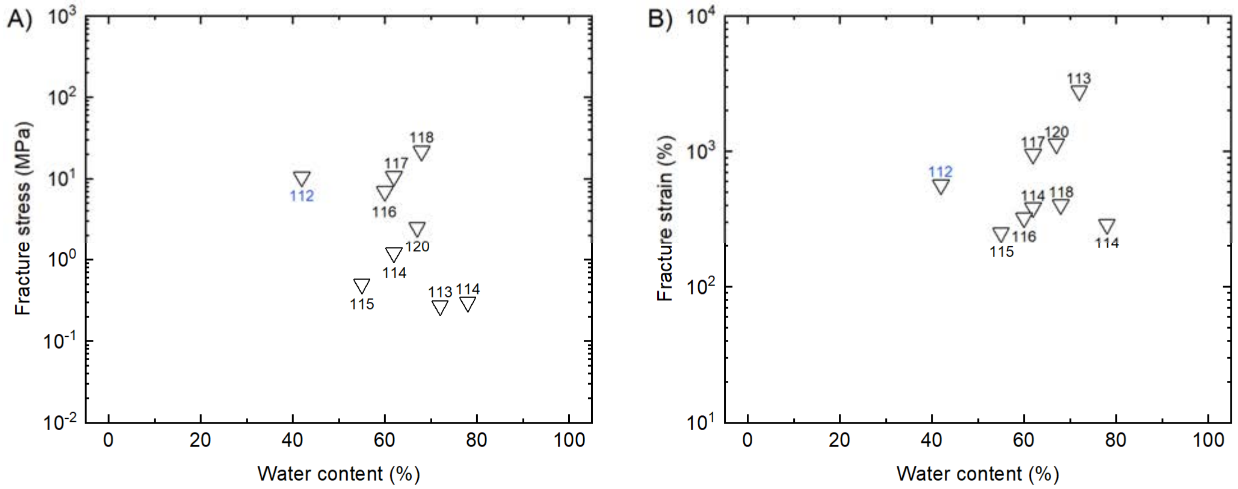

Figure S12. A) Fracture stress and B) fracture strain vs. water content for tough hydrogels through *hydrophobic interactions*. Labels refer to citations found in Table S1 (SN: black; DN: blue).

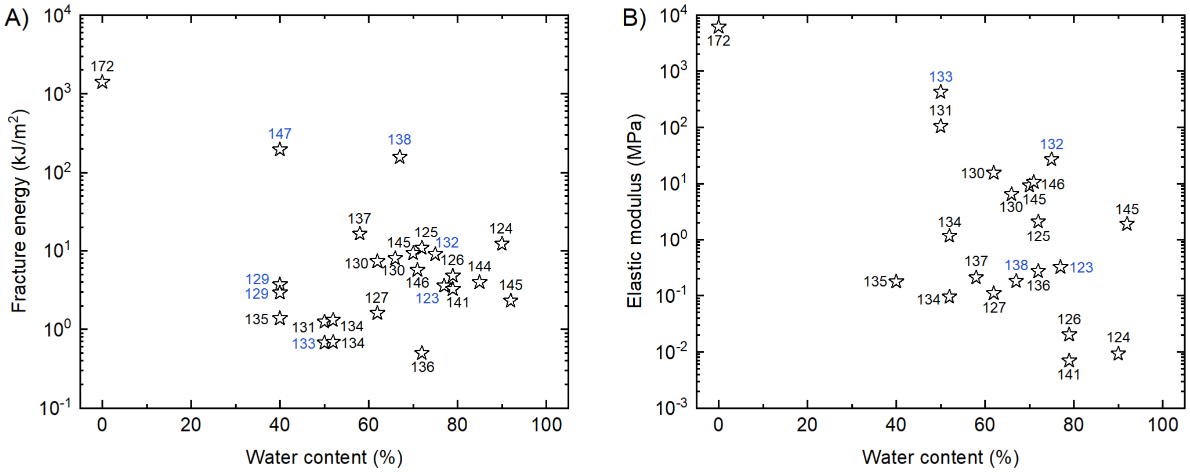

Figure S13. A) Fracture energy and B) elastic modulus vs. water content for tough hydrogels based on *(nano)composites*. Labels refer to citations found in Table S1 (SN: black; DN: blue).

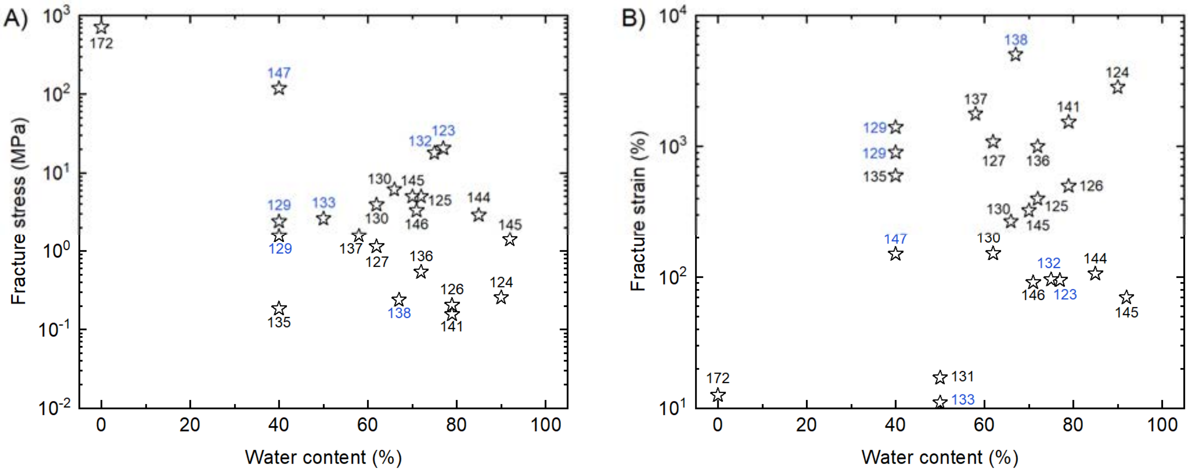

Figure S14. A) Fracture stress and B) fracture strain vs. water content for tough hydrogels based on *(nano)composites*. Labels refer to citations found in Table S1 (SN: black; DN: blue).

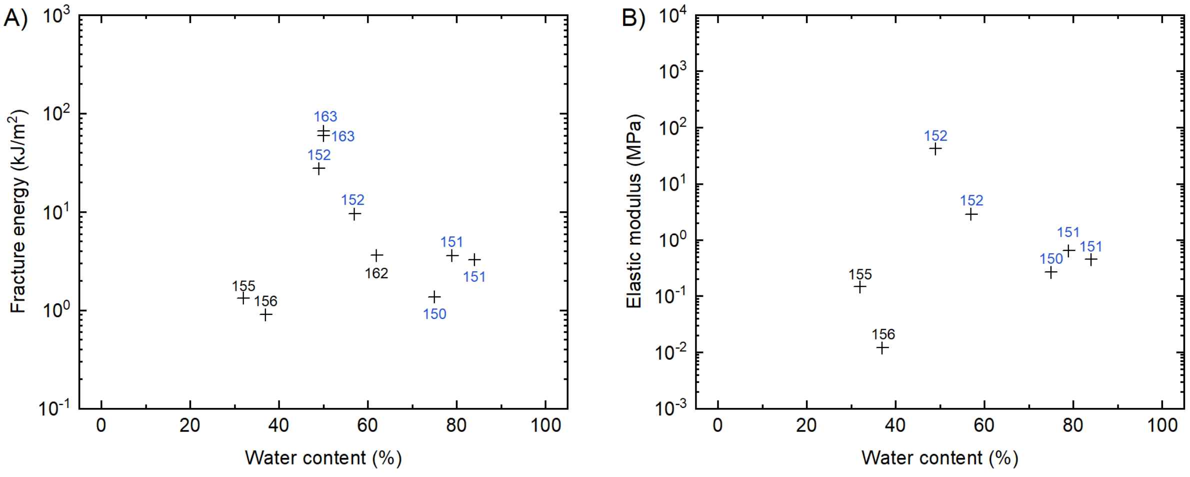

Figure S15. A) Fracture energy and B) elastic modulus vs. water content for tough hydrogels through *self-assembly*. Labels refer to citations found in Table S1 (SN: black; DN: blue).

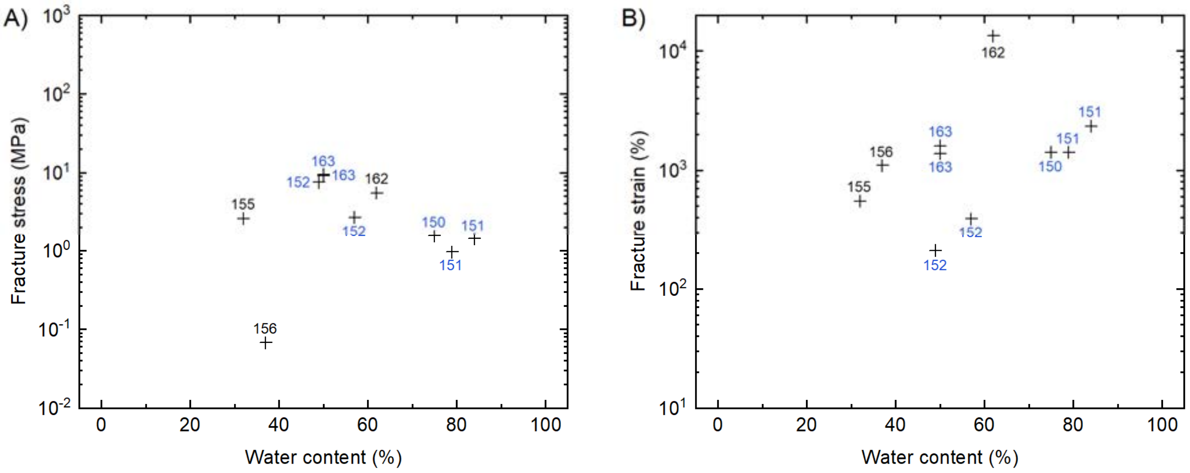

Figure S16. A) Fracture stress and B) fracture strain vs. water content for tough hydrogels based on *self-assembly*. Labels refer to citations found in Table S1 (SN: black; DN: blue)

3 Literature data

Table S1. Collected and extracted data from the literature for this review on tough hydrogels. Each entry is labeled according to the reference citation number in the main manuscript text. Categories are divided according to the individual mode of energy dissipation. Collected mechanical properties are subdivided into Tensile test (T), Fracture testing, Dissipation (referring to the hysteresis work), cyclic tensile tests (fatigue resistance), and self-recovery (mostly determining the hysteresis recovery).

REF	System							Tensile (T)								Fracture Testing						Dissipation		Cyclic Tensile / Fatigue Resistance					Self-Recovery					
		Category	Link	Components	Solvent	Sample		E	Slope	Dimensions	L_0	Strain rate	σ_b	ε_b	W	Γ	Notch	M	Dim.	Strain rate	Std.	U_{hys}	ε_{max}	M	Strain rate	Cycles	h_{RECOV}	ε_{max}	h_{RECOV}	time	h_{MAX}	time	ε_{max}	T
23	DN	metal-ligand	https://doi.org/10.1038/nature11409	PAAm/Alg–Ca^{2+}	H_2O	Hybrid Gel	86	0.029	5	75 x 5 x 3	?	2 min^{-1}	0.156	2300	-	9	40	PS	75 x 5 x 3	? ?	?	-	-	-	- -	-	-	-	-	-	-	-	-	-
35	DN	metal-ligand	https://doi.org/10.1021/mz5002355	PAAm/Alg–Ca^{2+}	H_2O	f = 33wt%	90	0.098	?	75 x 45 x 3	?	2 min^{-1}	0.25	1700	-	16	35	PS	75 x 45 x 3	2 min^{-1}	?	-	-	-	- -	-	-	-	-	-	-	-	-	-
36	DN	metal-ligand	https://doi.org/10.1002/adma.201801541	PAAm/Alg–Ca^{2+}	H_2O	30wt% CaCl2	78	?	?	50 x 10 x ?	?	1 min^{-1}	0.25	575	-	5.201	20	PS	?	? ?	?	-	-	-	- -	-	-	-	-	-	-	-	-	-
37	DN	metal-ligand	https://doi.org/10.1021/acsami.8b20520	P(AAm-*stat* -AAc)/Alg–Fe^{3+}	H_2O -><br>NaCl	s-Gels<br>(AA/AM=0.2)	51	24.6	5-15	20 x 4 x 1.5-2.5	?	100 mm min^{-1}	10.4	210	-	4.8	5	PS	40 x 15 x 1.5-2.0	100 mm min^{-1}	GB/T-528-2008	5	100	T	100 mm min^{-1}	?	?	?	53%	5 min	57%	30 min	100	25
38	DN	metal-ligand	https://doi.org/10.1016/j.jcis.2020.06.015	PAAm/Alg–Ca^{2+}	H_2O	H3	88.7	?	?	50 x 10 x 6.5	?	10 mm min^{-1}	0.12	>500	0.4382	0.177	20	Tr	50 x 10 x 6.5	10 mm min^{-1}	JIS-K6251	-	-	-	- -	-	-	-	-	-	-	-	-	-
39	DN	metal-ligand	https://www.science.org/doi/10.1126/sciadv.abl5066	PAAm/Alg–Ca^{2+}, trehalose	H_2O	30% Trehalose	64	?	?	25 x 5 x 2	?	0.2 s^{-1}	0.081	4100	-	5.116	5	Tr	25 x 5 x 2	? ?	?	-	-	-	- -	-	-	-	-	-	-	-	-	-
40	DN	metal-ligand	https://doi.org/10.3390/gels8050324	PNIPAAm, NaAlg, $CaSO_4$	H_2O	transitioned gel	?	?	?	75 x 5 x 3	?	2 min^{-1}	0.258	980	-	8.28	?	PS	?	? ?	?	-	-	-	- -	-	-	-	-	-	-	-	-	-
41	SN	metal-ligand	https://doi.org/10.1002/adma.201902381	Alg–Ca^{2+}	H_2O	C_{SA} = 15%	75	1.09	?	10 x ? x ?	?	20 mm min^{-1}	2.43	400	-	0.5	10	Tr	40 x 40 x 1.3-2	20 mm min^{-1}	?	-	-	-	- -	-	-	-	-	-	-	-	-	-
42	DN	metal-ligand	https://doi.org/10.1002/adhm.202001342	HA-Catechol, H_2O_2, Alg–Ca2+	H_2O	H4A7	?	?	?	20 x 10 x 1.5	?	2 mm min^{-1}	?	248	-	1.22	?	PS	?	? ?	?	-	-	-	- -	-	-	-	-	-	-	-	-	-
43	DN	metal-ligand	https://doi.org/10.1039/C4TB01289E	PAAm/κ/i-Carr–K^+/Ca^{2+}	H_2O	IC/PAAm gel	86	0.0072	?	30 x 8 x 2.5	?	50 mm min^{-1}	0.107	2520	-	9.5	?	?	?	? ?	?	-	-	T	10 mm min^{-1}	20	?	500	-	-	-	-	-	-
44	DN	metal-ligand	https://doi.org/10.1021/acsami.6b11363	PAAm/κ-Carr–K^+	H_2O	κ-Car/PAA,m DN HG (2:16)	82	0.13	?	? x 5 x 2.5	30	100 mm min^{-1}	0.558	2100	-	6.15	?	PS	?	? ?	?	12.6	?	T	100 mm min^{-1}	20'<br>90 °C	?	500	46%<br>76%	1000<br>10	?%<br>98%	?<br>20 min	500	25<br>90
45	DN	metal-ligand	https://doi.org/10.1021/acs.macromol.8b02269	PAAm/κ-Carr–Zr^{4+}	H_2O	DN-C_{30}-$C_{0.1}$-$C_{2.5}$-$C_{0.01}$	85	1.7	0-10	? X 2 x ?	12	100 mm min^{-1}	3.2	1870	-	18.5	10	Tr	35 x 12	100 mm min^{-1}	?	-	-	-	- -	-	-	-	-	-	-	-	-	-
46	DN	metal-ligand	https://doi.org/10.1039/D1SM01135A	PAAm/κ-Carr–K^+, gelatin	H_2O	35.09% Carr	82	0.0508	?	75 x 5 x 2.8		100 mm min^{-1}	0.122	5170	-	16.053	0-60	PS	?	100 mm min^{-1}	?	0.337	800	T	100 mm min^{-1}	20	10%	800	-	-	61%	30 min	800	60
47	SN	metal-ligand	https://doi.org/10.1021/acs.macromol.6b02150	P(AAm-*stat* -AAc)–Fe^{3+}	H_2O	AG-f15%	43	11.5	0-10	? X 2 x ?	12	100 mm min^{-1}	14.4	307	~40	~0.367	10	Tr	35 x 12	100 mm min^{-1}	?	-	-	T	100 mm min^{-1}	2	21%	250	47%	15	77%	4 h	250	25
48	SN	metal-ligand	https://doi.org/10.1016/j.polymer.2017.05.070	PAAc–Fe^{3+}, soak NaCl	H_2O	AAc 4M, NaCl 6M	52	0.376	5-10	40 x 10 x 2.6	?	50 mm min^{-1}	1.794	1321	11.5	1.637	20	Tr	40 x 10 x 2.6	50 mm min^{-1}	?	-	-	T	50 mm min^{-1}	-	-	-	-	-	-	-	-	-
49	SN	metal-ligand	https://doi.org/10.1016/j.mtphys.2020.100202	P(Aam-AAc)–Fe^{3+}	H_2O	fAAc = 10mol%<br>fAAc = 25mol%	<93	?	?	35 x 13 x 0.2	?	100 mm min^{-1}	3.2<br>7.8	430<br>250	1.5<br>7.6	1.3<br>2.191	5	PS	35 x 5 x 0.2	100 mm min^{-1}	?	4.48	420	T	100 mm min^{-1}	-	-	-	-	-	-	-	-	-
50	SN	metal-ligand	https://doi.org/10.1021/acsami.5b05074	P(VIm-*stat* -diaminotriazine)–Zn^{2+}	DMSO	PVV-4-10<br>PVV-2.5-5	76<br>77	20.58<br>11.41	?	30 x 4 x 0.2	?	100 mm min^{-1}	1.88<br>1.98	207<br>288	-	1.467<br>1.369	20	Tr	50 x 7.5 x 0.5	100 mm min^{-1}	GBT-529-2008A 1/2	-	-	-	- -	-	-	-	-	-	-	-	-	-
51	SN	metal-ligand	https://doi.org/10.1021/acsami.0c13654	P(AAm-*stat* -VIm)–Cu^{2+}	H_2O	PAV-3.5/2.5	44	15.4	10	35 x 6 x 1	10	100 mm min^{-1}	7.7	560	23.2	22.1	5	PS	15 x 1	100 mm min^{-1}	DIN-533504 S3	-	-	T	100 mm min^{-1}	5	?	500	-	-	-	-	-	-
52	SN	metal-ligand	https://doi.org/10.1002/adma.202005171	P(AAm-*stat* -AMPS)–Zr^{4+}	H_2O	TG-5%-0.1<br>TG-10%-0.1	74<br>54	0.4<br>2.2	10	? x 2 x 1	12	100 mm min^{-1}	2.3<br>5.3	544<br>995	-	0.2<br>4.6	10	Tr	35 x 2	100 mm min^{-1}	?	-	-	T	50 mm min^{-1}	-	-	-	-<br>77%	10 min	-<br>85%	30 min	100	10
54	SN	ionic	https://www.science.org/doi/10.1126/sciadv.aau3442	CS–phytate, gelatin	H_2O	C_4-G_{20}-P_{20}	51	2.47	?	25 x 2 x 2	?	50 mm min^{-1}	1.17	200	5.5	5.31	50%	PS	75 x 1 x 5	? ?	?	0.42	80	C	2 mm min^{-1}	5	69%	60	87%	5 min	100%	30 min	60	25
55	DN	ionic	https://doi.org/10.1039/d2ta06822b	PVA/CS–phytate	H_2O	PVA-CS-3PA-2	44	0.75	5-20	50 x 4 x 2	?	50 mm min^{-1}	6.02	915	30.48	5.28	20	Tr	50 x 7.5 x 2	50 mm min^{-1}	?	-	-	C	10 mm s^{-1}	20	?	70	-	-	-	-	-	-
56	DN	ionic	https://doi.org/10.1002/adma.201707071	PAAm/CS–cit^{3-}	H_2O	DN-Cit	56	1.3	?	35 x 5 x 2	?	50 mm min^{-1}	5.6	395	12.09	14	8	PS	20 x 2	? ?	?	9	350	C<br>T	10<br>100 mm min^{-1}	60<br>10	36%<br>32%	50<br>200	-	-	-	-	-	-
58	SN	ionic	https://doi.org/10.1038/nmat3713	P(NaSS-*stat* -MAPTAC)	H_2O	Cm 2.1-0.52	54	2.05	?	12 x 2 x 2-3	?	100 mm min^{-1}	1.82	742	7.12	4	20	PS/Tr	50 x 7.5 x 2-3	100 mm min^{-1}	JIS-K6251-7	-	-	T	100 mm min^{-1}	5	?	300	78%	10 min	100	120 min	300	25
59	SN	ionic	https://doi.org/10.1002/adma.201500140	PDMAEA-Q, PNaSS	H_2O	PDMAEA-Q/PNaSS	43	7.9	?	35 x ? x 2	12	100 mm min^{-1}	5.1	750	18.8	11.8	?	Tr	?	? ?	?	-	-	T	100 mm min^{-1}	?	?	?	61%	10 min	100	120 min	300	25
60	SN	ionic	https://doi.org/10.1021/acs.macromol.9b01676	P(NaSS-*stat* -MPTC), PEG	H_2O	PEG30%<br>ϕ = 56.6%	39	12.9	?	? x 2 x ?	12	100 mm min^{-1}	4.5	650	18.8	7.3	1	PS	20 x 10	100 mm min^{-1}	JIS-K6251-7	-	-	-	- -	-	-	-	-	-	-	-	-	-
61	SN	ionic	https://doi.org/10.1002/cplu.202000520	P(HEMA-DMAEMA*MeCl-SBMA),	H_2O	$H_2D_4S_4$	70	0.0017	?	3 (ϕ) x 40	10	100 mm min^{-1}	0.325	677	1	12	?	PS	?	? ?	?	-	-	T<br>C	100<br>10 mm min^{-1}	5 25	93%<br>88%	300<br>80	-	-	-	-	-	-
62	DN	ionic	https://doi.org/10.1021/acs.jpclett.0c02116	PMPTC/PAAc	H_2O	Post-C-0<br>Post-C-2	60	?	?	30 x 10	?	100 mm min^{-1}	0.06<br>0.7	70<br>190	-	0.266<br>4.686	?	PS	?	? ?	?	0	100	T	100 mm min^{-1}	?	?	?	-	-	-	-	-	-
63	DN	ionic	https://doi.org/10.1021/acsami.8b10280	PAAm/CS–Fe^{3+}, polypyrrole	H_2O	PAM/$CS_{0.75}$-PPy20%	75/<br>~40?	50.1	?	25 x ? x 3	5	2 mm s^{-1}	0.8	260	-	12	5	PS	25 x ? x 3	2 mm s^{-1}	?	-	-	-	- -	-	-	-	-	-	-	-	-	-
64	DN	ionic	https://doi.org/10.1039/D0MH01231A	P(AAc-*stat* -BnDiol)/CS	H_2O	P(AA-co-$UCAT_5$)-CS_3	72 -<br>74	?	?	25 x 4 x 4	?	100 mm min^{-1}	0.57	2514	-	6.625	5	Tr	40 x 20 x 4	50 mm min^{-1}	?	-	-	-	- -	-	-	-	-	-	-	-	-	-
65	SN	ionic	https://doi.org/10.1021/acsami.1c04781	PAAm, carboxymethyl CS	H_2O	EHG-3-70-30	47	0.119	20	12 x 2	?	100 mm min^{-1}	2	2196	11	2.175	10	Tr	40 x 15	100 mm min^{-1}		0.115	500	T	100 mm min^{-1}	10	48%	500	?	?	100%	2 min	500	25

Table S1. continued.

69	SN	H-bonding	https://doi.org/10.1039/D0SM00981D	PAAm	H_2O		71<br>56	0.0063<br>0.028	10	10 x ? x ?	5	0.1 s^{-1}	0.06<br>0.1	1300<br>400	-	1.85<br>1.75	0,3,6	?	?	? ?	?	-	-	-	- -	-	-	-	-	-	-	-	-	-
70	SN	H-bonding	https://doi.org/10.1002/adma.201503724	P(DMAAm-*stat*-MAAc)	H_2O	D40-I0.5	67	28	?	50 x 6 x 1.6	18	0.083 s^{-1}	1.6	800	-	9.3	20	Tr	50 x 7.5	5 min^{-1}	DIN 53504-S	-	-	T	0.083 s^{-1}	10	46%	300	?	?	100	60 min	300	22
71	SN	H-bonding	https://doi.org/10.1021/acs.chemmater.8b05262	P(MAAm-*stat*-PMAAc)	H_2O	MM-0.3-6<br>MM-0.3-3.5	46<br>70	217.3<br>11.5	8	12 x 2 x 1	?	100 mm min^{-1}	8<br>1.2	418<br>198	0.028<br>0.002	23.5<br>2.9	10	Tr	35 x 8 mm	100 mm min^{-1}	?	-	-	-	- -	-	-	-	-	-	-	-	-	-
72	SN	H-bonding	https://doi.org/10.1021/acs.biomac.0c01788	P(AAm-*stat*-PheAcr)	H_2O	$PAPA_{0.02}$-PAM_3	82	?	?	16 x 4 x 2	?	50 mm min^{-1}	0.157	2136	-	1.134	8	PS	20 x 2	? ?	?	0.005<br>0.034	80<br>1000	C<br>T	10 mm min^{-1}	100<br>10	89%<br>31%	80	67%	10 min	85%	20 min	500	25
73	SN	H-bonding	http://dx.doi.org/10.1039/C9TC04945B	P(NPAm-*stat*-MAAc)	DMSO	MN4<br>MN10	70<br>51	1.8<br>66	5	50 x 7.5 x 1.2	?	120 mm min^{-1}	2.2<br>6.3	460<br>570	5.8<br>(MN6)	0.5<br>7	20	Tr	7.5 x 50 x 1.2	240 mm min^{-1}	GBT 529-2008 A	5.2<br>(MN6)	300	T	120 mm min^{-1}	?	?	?	62%	10 min	96%	90 min	200	25
74	SN	H-bonding	https://doi.org/10.1016/j.polymer.2017.09.044	P(VIm-*stat*-AAc)	DMSO -><br>H2O	HG-20 (C=3M)	35	0.7	0-10	? x ? x 2	12	100 mm min^{-1}	1.8	1090	-	5.6	10	Tr	35 x 8	100 mm min^{-1}	?	-	-	T	100 mm min^{-1}	2	?	300	91%	10 min	-	-	300	25
75	SN	H-bonding	https://doi.org/10.1021/acs.macromol.8b01496	P(MAAc-*stat*-VIm)	DMSO	VM-3-20-1<br>VM-5-10-1	50 -60	80<br>175	10	l x w x 2	12	100 mm min^{-1}	20<br>40	40<br>117	-	4.5<br>-	10	Tr	35 x 8 mm	100 mm min^{-1}	?	-	-	-	- -	-	-	-	-	-	-	-	-	-
76	SN	H-bonding	https://doi.org/10.1002/adma.201500534	P(AAm-*stat*-NAGA)	H_2O	PNAGA-25	75	0.137	?	50 x 6 x 0.5	?	50 mm min^{-1}	1.1	1400	-	1.1	20	Tr	50 x 7.5 x 0.5	50 mm min^{-1}	GBT 529-2008 A	-	-	T	50 mm min^{-1}	?	?	?	60%	10 min	~95%	24 h	400	25
77	SN	H-bonding	https://doi.org/10.1002/marc.201800819	PNAGA hyperbranched	DMSO -><br>H_2O	L-PNAGA-35<br>HB-PNAGA-35	65<br>65	0.406<br>1.172	?	50 x 8 x 1	?	50 mm min^{-1}	1.879<br>2.724	1034<br>902	-	1.778<br>2.205	20	Tr	50 x 8 x 1	50 mm min^{-1}	GBT-529-2008A	-	-	-	- -	-	-	-	-	-	-	-	-	-
78	SN	H-bonding	https://doi.org/10.1039/C9MH01844A	P(NASC)	H_2O	PNASC-15<br>PNASC-30	61<br>46	48.4<br>100.3	?	?	?	50 mm min^{-1}	1.7<br>4.7	341<br>638	5.66<br>20.35	5.65<br>11.4	20	Tr	?	50 mm min^{-1}	ASTM standard	2.33	100	T	50 mm min^{-1}	?	?	?	-	-	-	-	-	-
79	SN	H-bonding	https://doi.org/10.1002/adfm.202210395	P(NASC-*stat*-AAc)	DMSO -><br>H2O	$PNA_{1/0.4}$	35	0.2	1-5	30 x 5 x 0.8-1	?	100 mm min^{-1}	5.8	983	34	144	20	Tr	40 x 10	100 mm min^{-1}	?	0.7	100	T	100 mm min^{-1}	100	17%<br>(10)	100	-	-	-	-	-	-
80	SN	H-bonding	https://doi.org/10.1021/acsabm.8b00546	hydrophilic PU–lignin	H_2O	2.5 wt %<br>lignin:HPU	76	2.62	?	15 x 5	?	15 mm min^{-1}	1.86	225	-	2.05	20	Tr	15 x 5	15 mm min^{-1}	?	-	-	T	15 mm min^{-1}	10	30%	100	-	-	-	-	-	-
81	SN	H-bonding	https://doi.org/10.3390/polym12010239	hydrophilic PU–tannic acid	DMF -><br>H_2O	TA10-PU(S)	<80	0.15	5-10	50 x 10 x 1	20	100 mm min^{-1}	0.75	350	-	2.2	0,3,6	Tr	?	100 mm min^{-1}	GBT-529-2008A	0.15	200	-	- -	-	-	-	-	-	-	-	-	-
82	SN	H-bonding	https://pubs.acs.org/doi/10.1021/acsbiomaterials.0c00915?ref=pdf	P(LGA-*g*-EGA), gellan gum	H_2O	PLGA/GG	85	0.08	?	30 x 7.8 x 1.5	?	0.5 % min^{-1}	0.027	40	-	7.7	?	?	?	? ?	?	-	-	C	10 % min^{-1}	50	?	40	-	-	-	-	-	-
83	SIPN	H-bonding	https://doi.org/10.1039/D0SM00422G	PMAAc, Gelatin	H_2O	G-M8-8C<br>G-M8-2C	55<br>45	5.34<br>11.3	?	12 x 2 x 1	12	0.14 s^{-1}	1.39<br>4.93	296<br>481	-	3.65<br>8.52	10	Tr	35 x 15	100 mm min^{-1}	JIS-K6215-7	-	-	T	0.14 s^{-1}	?	?	?	61%	5 min	100%	40 min	100	25
89	SN	crystallization	https://doi.org/10.1039/C2SM26102B	PVA	H_2O	DFT-10-7 //<br>DFT-10-7 _I	<90	0.07<br>0.09	10-30	75 x 10(4) x 2	20	800 mm min^{-1}	0.47<br>0.69	241<br>287	-	0.265<br>0.427	20	Tr	50 x 7.5 x 4	10 mm min^{-1}	JIS-K6252 1/2	-	-	T	400 mm min^{-1}	10	?	100	-	-	-	-	-	-
90	SN	crystallization	https://doi.org/10.1038/s41586-021-03212-z	PVA–cit^{3-}	H_2O	HA-5PVA	90	1.98	?	l x w x 2	2	10 % s^{-1}	11.5	2900	175	131	8	PS	40 x 20 x 1-2	10 % s^{-1}	?	-	-	T	? mm min^{-1}	10	?	500	-	-	-	-	-	-
91	SN	crystallization	https://doi.org/10.1126/sciadv.aau8528	PVA	H_2O	90 min annealed	60	9.8	?	? x 5 x 0.8	10	50 mm min^{-1}	8.8	360	-	8.9		Tr		? ?	?	-	-	-	- -	-	-	-	-	-	-	-	-	-
92	SN	crystallization	https://doi.org/10.1016/j.jiec.2022.05.034	PVA, graphene oxide, tannic acid	H_2O	s-PVA/1% GO-TA	<70	?	?	75 x 4 x 2	?	50 mm min^{-1}	14.38	450	27.93	3.69	10	Tr	40 x 12	? ?	GB/T 529-2008	2.18	150	T	100 mm min^{-1}	-	-	-	-	-	-	-	-	-
93	SN	crystallization	https://doi.org/10.1016/j.jiec.2022.05.035	PVA, PEG-diepoxide	H_2O	4% epoxy	80	0.126	?	? x 4 x 3	20	40 mm min^{-1}	1.1	600	2.9	0.39	20	Tr	50 x 14 x 5	40 mm min^{-1}	?	0.019	100	T	40 mm min^{-1}	5	28%	100	-	-	-	-	-	-
94	SIPN	crystallization	https://doi.org/10.1016/j.jiec.2022.05.036	PVA, HA, PEG, collagen	H_2O	TEHy-6	80	25.4	?	? x 20 x 5	20	20 mm min^{-1}	23	120	1.17	1.75	?	?	?	? ?	?	-	-	-	- -	-	-	-	-	-	-	-	-	-
95	DN	crystallization	https://doi.org/10.1016/j.jiec.2022.05.037	PVA/PAAm	H_2O	PVA-PAAm 25%	62	5	?	75 x 45 x 3	?	2 min^{-1}	2.5	285	-	14	35	?	75 x 20 x 3	2 min^{-1}	?	-	-	-	- -	-	-	-	-	-	-	-	-	-
96	DN	crystallization	https://doi.org/10.1016/j.jiec.2022.05.038	PVA/Alg–Ca^{2+}	H_2O	PVA/Alg 60/1	>77	0.257	?	40-50 x 5 x 3	25	50 mm min^{-1}	1.9	510	4.461	0.453	10	?	40 x 10 x 3	50 mm min^{-1}	?	-	-	T	1 mm min^{-1}	-	-	-	52%	10 min	~76%	60 min	100	25
97	DN	crystallization	https://doi.org/10.1016/j.jiec.2022.05.039	PVA/Alg–Ca^{2+}	H_2O	Alg/PVA-0.5<br>Alg/PVA-2	~76<br>45	0.051<br>3.71	?	? x 2 x 1.2-2.6	12	100 mm min^{-1}	1.35<br>15	683<br>765	3.32<br>55.98	2.45<br>39.57	10	PS	60 x 40 x 1.2-2.6	100 mm min^{-1}	?	0.71<br>3.40	450	T	? mm min^{-1}	-	-	-	-	-	-	-	-	-
98	DN	crystallization	https://doi.org/10.1016/j.jiec.2022.05.040	PVA/pectin–Ca^{2+}	H_2O	PVA8-CaP	65	0.2	?	10 x 3.2 x 1-1.5	?	50 mm min^{-1}	1.7	240	-	0.9	>5	Tr	20 x 10 x 0.66-0.85	20 mm min^{-1}	?	-	-	T	50 mm min^{-1}	10	?	100	-	-	-	-	-	-
99	DN	crystallization	https://doi.org/10.1016/j.jiec.2022.05.041	PVA/PAAc–Fe^{3+}	H_2O	PVA/PAA-48 14%<br>cold-drawn n=240%	36	7.1<br>100	10-30	?	5	100 mm min^{-1}	20.7<br>140	1080<br>135	117<br>110	101<br>-	5	PS	15 x ? x 1	100 mm min^{-1}	?	-	-	T	100 mm min^{-1}	-	-	-	-	-	-	-	-	-
102	DN	salting-out	https://doi.org/10.1016/j.jiec.2022.05.042	PAAm/CS–soak X	H_2O	PAM-CS-A<br>PAM-CS-S	80	0.319<br>0.358	5-15	35 x 5 x 2	5	50 mm min^{-1}	2.12<br>1.94	470<br>560	3.78<br>3.47	12.9<br>8.3	8	PS	20 x 10 x 2	50 mm min^{-1}	?	3.26<br>2.97	300	T	100 mm min^{-1}	10	27%<br>24%	200	65%<br>65%	15 min	95%<br>90%	4 h	200	25
103	DN	salting-out	https://doi.org/10.1016/j.jiec.2022.05.043	PAAc/CS–soak NaCl	H_2O	PAA-$FE^{3+}_{0.6}$/CS-6h	?	?	?	25 x 4 x ?	10	100 mm min^{-1}	3.7	1200	9.81	2.8	2	PS	?	100 mm min^{-1}	?	6.86<br>0.087	800<br>200	T	150 mm min^{-1}	-<br>10	-<br>29%	800<br>200	-	-	-<br>>100	- 24<br>h	-<br>200	-<br>25
104	DN	micellization	https://doi.org/10.1016/j.jiec.2022.05.044	P(AAm-*stat*-SMA)–SDS/Agar/soak NaCl	H_2O	$A_{20}H_{1.0}$-Am_{30}	75	0.106	?	8.5 (ϕ) x 60	?	100 mm min^{-1}	0.267	526	9.35	1	20	Tr	40 x 10 x 10	50 mm min^{-1}	?	0.503	1000	T	100 mm min^{-1}	6	16%	1000	51%	10 min	52%	120 min	1000	25
105	DN	micellization	https://doi.org/10.1016/j.jiec.2022.05.045	P(AAm-*stat*-SMA)–SDS/Agar/soak LiCl	H_2O	P(Aam-SMA) 6<br>P(Aam-SMA) 12	50<br>29	0.011<br>0.012	0-10	? X 2 x 2	12	100 mm min^{-1}	0.215<br>0.216	2150<br>1660	-	4.5<br>6.834	?	PS	?	100 mm min^{-1}	?	-	-	-	- -	-	-	-	-	-	-	-	-	-
106	sIPN	micellization	https://doi.org/10.1016/j.jiec.2022.05.046	PAAm, P(SA-*stat*-MAAc)–CTAB	H_2O	C1/10-SM14<br>C1/6-SM3	90<br>54	1.31<br>9.25	?	35 x 2 x 2	20	100 mm min^{-1}	1.32<br>2.73	717<br>1149	5.73<br>22.31	0.6<br>14.581	20	Tr	50 x 7.5 x 2	100 mm min^{-1}	JIS-K6252	-	-	-	- -	-	-	-	-	-	-	-	-	-
107	DN	micellization	https://doi.org/10.1016/j.jiec.2022.05.047	P(AAc-*stat*-LMA)–CTAB/PANI	H_2O	PAAN<br>PAAN/Gly-24	?	0.048<br>0.128	0-25	50 x 4 x 2	20	100 mm min^{-1}	0.81<br>1.38	2378<br>2151	6.62<br>15.66	4.503<br>13.987	?	PS	?	100 mm min^{-1}	?	0.140	400	T	100 mm min^{-1}	-	-	-	55%	5 min	89%	30 min	400	25
108	SN	micellization	https://doi.org/10.1016/j.jiec.2022.05.048	PAAm, κ-casein	H_2O	α = 32%	77	<0.012	?	76 x 5 x 3	?	6 min^{-1}	0.035	3500	-	3	15/30/45	?	?	6 min^{-1}	?	-	-	-	- -	-	-	-	-	-	-	-	-	-
109	SN	micellization	https://doi.org/10.1016/j.jiec.2022.05.049	Pluronics F127 DA, HA-MA	H_2O	NMGe15%	85	?	0-25	?	?	? ?	-	-	-	1.204	?	?	?	? ?	?	-	-	-	- -	-	-	-	-	-	-	-	-	-
110	SN	micellization	https://doi.org/10.1016/j.jiec.2022.05.050	PU Acrylate μ–εmulsion, P(AAm-*co*-IBOA-*co*-AN)	H_2O	WPUA-4AAm-5IBOA<br>WPUA-3AAm-6AN	23	0.033<br>0.076	?	50 x 4 x 1	?	100 mm min^{-1}	12.5<br>24.7	724<br>544	39.8<br>68.9	-<br>37.2	20	Tr	50 x 7.5 x 2	100 mm min^{-1}	JIS-K6252 1/2	-	-	-	- -	-	-	-	-	-	-	-	-	-
111	SN	micellization	https://doi.org/10.1016/j.jiec.2022.05.051	P(Aam-*co*-DVB)–SDS	H_2O	$PAAM$-$DVB_{0.08}$	86	0.090-<br>0.357	?	50(16) x 8.5(4) x 2	10	100 mm min^{-1}	1.5	10200	18.8	26	25	Tr	? x 50 x 1.5	100 mm min^{-1}	?	0.112	700	T	100 mm min^{-1}				-	-	~100%	3 min	700	25

Table S1. continued.

112	DN	hydrophobic	https://doi.org/10.1016/j.jiec.2022.05.052	P(BuMA-*b*-AAc-*b*-BuMa)/PAAm	H_2O	B-DN3	42	2.2	?	35 x 2 x 1	12	0.14 s^{-1}	10.5	570	-	2.85	10	Tr	40 x 20 x 0.71	100 mm min^{-1}	?	-	-	T	100 mm min^{-1}	?	?	100	85%	5 min	100%	16 min (heating /cooling)	100	25
113	DN	hydrophobic	https://doi.org/10.1016/j.jiec.2022.05.053	P(AA-*stat*-OMA)/xanthan gum–Ca^{2+}	H_2O	DICH-2	72	?	?	10 x 5 x 2	?	20 mm min^{-1}	0.27	2800	4.82	1.9	?	?	?	? ?	?	-	-	T	? ?	5	?	300	-	-	-	-	-	-
114	SN	hydrophobic	https://doi.org/10.1016/j.jiec.2022.05.054	PHU, P(SA-*stat*-AAc)	EtOH -> H_2O	C_{PHU} 0%<br>C_{PHU} 20%	78<br>62	0.2<br>0.7	0-10	50 x 10 x ?	30	100 mm min^{-1}	0.3<br>1.2	290<br>386	-	1.248<br>2.632	10	Tr	35 x 8 x ?	100 mm min^{-1}	?	-	-	-	- -	-	-	-	-	-	-	-	-	-
115	SN	hydrophobic	https://doi.org/10.1016/j.jiec.2022.05.055	P(NIPAAm-*stat*-FOSA)	bulk	NF8 15°C	55	1.57	?	?	?	50 mm min^{-1}	0.496	251	-	3.8	?	PS	?	50 mm min^{-1}	?	1.16	400	T	50 mm min^{-1}	5	18%	400	?	12 min	91%	60 min	200	5
116	SN	hydrophobic	https://doi.org/10.1016/j.jiec.2022.05.056	P(AAm-*stat*-PhA)	DMSO -> H2O	P(PA-co-Aam)(2:2)	<60	145	?	40 x 5 x 1	10	500 % min^{-1}	6.92	322	20.65	7.453	40%	PS	10 x 5/10/20 x 1	500 % min^{-1}	?	-	-	T	100 % min^{-1}	10		20	60%	30 min	-	-	20	25
117	SN	hydrophobic	https://doi.org/10.1016/j.jiec.2022.05.057	P(AN-*co*-AAm-*co*-PEGDMA), dimethylanilin	DMSO -> H2O	RAN 66%	62	15.97	?	? x 2 x 0.5	10	0.17 s^{-1}	10.6	950	-	16.596	20	Tr	50 x 7.5 x 0.5	100 mm min^{-1}	GBT 529-2008 A	-	-	-	- -	-	-	-	-	-	-	-	-	-
118	SN	hydrophobic	https://doi.org/10.1016/j.jiec.2022.05.058	P(AN-*stat*-AAc)–Zn^{2+}	DMSO -> H2O	P(AN/AANa)-13H<br>P(AN/AA0-15-35	68	17.4	?	20 x 2 x 0.5	?	100 mm min^{-1}	21.85	404	50.8	7.053	40	Tr	100 x 15 x 0.5	100 mm min^{-1}	GB/T 528-2009	-	-	T	100 mm min^{-1}	10	?	100	-	-	-	-	-	-
119	SN	hydrophobic	https://doi.org/10.1016/j.jiec.2022.05.059	Polyether-based PU	EtOH -> H2O	0% Urea<br>50% Urea	50<br>66	1.2<br>0.3	?	25 x 4 x ?	25	50 mm min^{-1}	-	-	-	2.8<br>0.75	20	Tr	?	50 mm min^{-1}	?	-	-	-	- -	-	-	-	-	-	-	-	-	-
120	SN	hydrophobic	https://doi.org/10.1016/j.jiec.2022.05.060	PDMAAm–lignin	H_2O	DL_8:11	67	2.5	?	? X 2 x 0.7	12	0.14 s^{-1}	2.5	1130	-	16	10	Tr	35 x 15	100 mm min^{-1}	JIS-K6215-7	?	500	T	0.14 s^{-1}	?	?	?	14%	10 min	-	-	500	25
123	DN	nanospheres	https://doi.org/10.1016/j.jiec.2022.05.061	PAAm/agar/chondroitin sulfate-coated SiO_2	H_2O	CS-DN as prep	77	0.3213	10	Compressive			20.4	94.2	-	3.544	Compressive			? ?	?	0.00146	90	C	? mm min^{-1}	5	50%	90	-	-	-	-	-	-
124	SN	nanospheres	https://doi.org/10.1016/j.jiec.2022.05.062	P(AAm-*stat*-SMA), vinyl SO_2	H_2O	TC-NCP gel-5%	90	0.0092	?	5 (ϕ) x 60	15	100 mm min^{-1}	0.256	2820	1.93	12.1	7.5	PS	40 x 25 x 2	100 mm min^{-1}	?	0.133	2000	-	- -	-	-	-	-	-	-	-	-	-
125	SN	nanospheres	https://doi.org/10.1016/j.jiec.2022.05.063	PVA, SiO_2	H_2O	PVA-90-12	72	2.1	5-15	20 x 10 x 0.96	?	100 mm min^{-1}	5	398	-	10.7	4<br>8	PS/Tr	20 x 10 x 0.96	100 mm min^{-1}	?	8.6<br>-	400<br>48	T<br>C	10 mm min^{-1}	-<br>80	-<br>?	400<br>48	-	-	-	-	-	-
126	SN	nanospheres	https://doi.org/10.1016/j.jiec.2022.05.064	PNIPAAm, silsesquioxane	H_2O	7% -- M0.01	79	0.02	?	6.5 (ϕ) x 50	10	10 mm min^{-1}	0.204	500	-	4.781	?	?	?	? ?	?	-	-	T	10 mm min^{-1}	5		300	-	-	-	-	-	-
127	SN	nanospheres	https://doi.org/10.1016/j.jiec.2022.05.065	PAMPS, PAH, Ag NPs	H_2O	NP5-PAMSPEG-20	62	0.11	?	30 x 9 x 2	?	50 mm min^{-1}	1.14	1085	-	1.6	?	?	?	? ?	?	0.014	90	C	1 mm min^{-1}	5	71% (5th)	90	-	-	-	-	-	-
128	DN	nanospheres	https://doi.org/10.1016/j.jiec.2022.05.066	P(Aam-*stat*-AAc)/CS/Fe_3O_4	H_2O	Aam-CS-Fe DN	?	0.16	5-15	35 x 5 x 2	?	50 mm min^{-1}	0.158	419	-	9.41	5	PS	?	100 mm min^{-1}		-	-	-	- -	-	-	-	-	-	-	-	-	-
129	DN	nanospheres	https://doi.org/10.1016/j.jiec.2022.05.067	PHEAA/gelatin, V-ZIF-8 NP	H_2O	V-ZIF-8 0.1 wt% V-ZIF-8 1.0 wt%	<40	?	?	? x 3.18 x 1	25	100 mm min^{-1}	2.4<br>1.56	1400<br>900	14.1<br>6.6	2.9<br>3.7	?	?	?	? ?	ASTM-638-V	2.75<br>?	1100<br>?	T	100 mm min^{-1}	?	?	?	-	-	-	-	-	-
130	SN	nanospheres	https://doi.org/10.1016/j.jiec.2022.05.068	P(AN-*stat*-VIm), CaP	DMSO -> H_2O	PAV-10%-CaP PAV-20%-CaP	66<br>62	6.47<br>15.38	?	30 x 4 x 0.2	?	100 mm min^{-1}	6.1<br>3.9	269<br>151	-	7.935<br>7.292	20	Tr	50 x 7.5 x 0.5	100 mm min^{-1}	GBT-529-2008A 1/2	-	-	-	- -	-	-	-	-	-	-	-	-	-
131	SN	nanospheres	https://doi.org/10.1016/j.jiec.2022.05.069	PAAm, CaP	?	PAAm-/-MBAm-3	50	103	?	?	?	? mm min^{-1}	?	17	-	1.246	?	?	?	? mm min^{-1}	?	-	-	-	- -	-	-	-	-	-	-	-	-	-
132	DN	nanospheres	https://doi.org/10.1016/j.jiec.2022.05.070	PVA/Alg, CaP	H_2O	PSH200%	75	26.93	?	?	?	50 mm min^{-1}	17.84	96	10.47	8.97	5	PS	40 x 20 x 0.2	50 mm min^{-1}	?	-	-	-	- -	-	-	-	-	-	-	-	-	-
133	DN	nanospheres	https://doi.org/10.1016/j.jiec.2022.05.071	P(DMAAm-*co*-AAm-*co*-AAc), $CaCO_3$	H2O	35% Aac	50	420	?	20 x 5 x 0.4-0.8	10	5 % min^{-1}	2.59	11	-	0.673	2.5	PS	20 x 5 x 0.4-0.8	5 % min^{-1}	?	-	-	-	- -	-	-	-	-	-	-	-	-	-
134	SN	nanospheres	https://doi.org/10.1016/j.jiec.2022.05.072	PAAm, $CaCO_3$	H_2O	0 wt% AA<br>15 wt% AA	52	0.094<br>1.146	?	?	20	100 mm min^{-1}	?	?	?	1.304<br>0.686	7.5	PS	30 x 15 x 2	100 mm min^{-1}	?	-	-	-	- -	-	-	-	-	-	-	-	-	-
135	SN	nanospheres	https://doi.org/10.1016/j.jiec.2022.05.073	PHEMA, Biochar	EtOH -> H2O	3-biochar/PHEMA	40	0.178	?	15 x 2 x 2	15	30 mm min^{-1}	0.183	600	-	1.368	20	PS	70 x 30 x 2	? mm min^{-1}	?	-	-	-	- -	-	-	-	-	-	-	-	-	-
136	SN	nanospheres	https://doi.org/10.1016/j.jiec.2022.05.074	PAAm, P(BuA-*stat*-St) microspheres	H_2O	$C_{peroxide}$ 1.37, C_M 5M	72	0.27	10-30	75 x 4 x 2	20	80 mm min^{-1}	0.54	1000	-	0.5	20	Tr	50 x 7.5 x 5	10 mm min^{-1}	JIS-K6252 1/2	-	-	T	80 mm min^{-1}	10	?	300	-	-	-	-	-	-
137	SN	nanospheres	https://doi.org/10.1016/j.jiec.2022.05.075	PAAm, PAMPS micropheres, NdFeB NPs, Laponite XLG	H_2O	10% NdFeB	58	0.21	?	? x 2 x 1	12	100 mm min^{-1}	1.58	1770	-	16.4	20	PS	50 x 30	30 mm min^{-1}	?	-	-	-	- -	-	-	-	-	-	-	-	-	-
138	DN	nanospheres	https://doi.org/10.1016/j.jiec.2022.05.076	PAAm, Span 80, PAMPS microspheres	H_2O	PAMPS@PAAm 3.6% CL	67	0.18	?	100 x 2 x	?	50 mm min^{-1}	0.237	5000	7.85	157	2	PS	?	? ?	?	-	-	-	- -	-	-	-	-	-	-	-	-	-
139	SN	nanosheets	https://doi.org/10.1016/j.jiec.2022.05.077	PAAm, Laponite XLG, TiO_2	H_2O	MLT0.2-1	?	-	-	-	-	- -	-	-	-	1.837	?	?	?	? ?	?	-	-	C	? mm min^{-1}	10	?	60	?	?	?	?	60	25
140	DN	nanosheets	https://doi.org/10.1016/j.jiec.2022.05.078	P(NIPAAm-*stat*-OEGMA)/PANI, Laponite XLS	H_2O	PANI-6/PMON<br>PANI-8/PMON	?	11.4<br>24.41	0-10	?	10	50 mm min^{-1}	~6.85<br>7.21	356<br>153	-	172<br>85.7	?	?	?	50 mm min^{-1}	?	-	-	T	50 mm min^{-1}	300	?	50	-	-	-	-	-	-
141	SN	nanosheets	https://doi.org/10.1016/j.jiec.2022.05.079	P(AAm-Isocyanatoethyl GlnMA), Laponite XLG	H_2O	$L_5A_3G_{0.11}$	79	0.007	?	40 x 6 x 2	?	50 mm min^{-1}	0.156	1539	-	3.25	8	PS	20 x 2	50 mm min^{-1}	0.868	0.010<br>0.045	200<br>1000	T	50 mm min^{-1}	10	35%	200	82%	10 min	85%	20 min	200	25
144	SN	nanosfibers	https://doi.org/10.1016/j.jiec.2022.05.080	Alg–Ca^{2+}, gelatin NF mats	H_2O	angle-ply / cross/ply	85	?	?	l x 5 x d	20	0.5 mm s^{-1}	2.9	106	-	3.96	3	Tr	15 x 10	0.5 mm s^{-1}	?	-	-	-	- -	-	-	-	-	-	-	-	-	-
145	SN	nanofibers	https://doi.org/10.1016/j.jiec.2022.05.081	PVA,polyaramid NF	DMSO -> H2O	ANF-PVA 8<br>ANF-PVA 30	92<br>70	1.9<br>9.1	0-5	?	?	0.2 % s^{-1}	1.4<br>5.0	70<br>325	-	2.3<br>9.2	?	PS	?	? % s^{-1}	?	-	-	T	? mm min^{-1}	5	-	20	-	-	-	-	-	-
146	SN	nanofibers	https://doi.org/10.1016/j.jiec.2022.05.082	PVA, polyaramid NF, Ag	DMSO	ANF1.96%/PVA/AgNWs	71	10.7	?	35 x 6 x 0.32	?	10 mm min^{-1}	3.3	91	-	5.7	?	PS	?	? ?	?	-	-	-	- -	-	-	-	-	-	-	-	-	-
147	DN	nanofibers	https://doi.org/10.1016/j.jiec.2022.05.083	PVA/PAAm, Kevlar/Nomex	H_2SO_4	PF Type A seawater soak	40-70	?	?	?	?	0.01 s^{-1}	118.6<br>103.5	150	230<br>307	195.8<br>-	?	both	60 x 20/40/60	50 mm min^{-1}	?	-	-	T	? mm min^{-1}	1000	?	120	-	-	-	-	-	-
148	DN	nanofibers	https://doi.org/10.1016/j.jiec.2022.05.084	PAAm/Alg–Ca2+, glass fibers	H_2O	Hydrogel Composite	?	35	5	40 x 3 x ?	10	30 mm min^{-1}	2.4	58	-	206.7	20	Tr	50 x 45 x ?	30 mm min^{-1}	?	0.16	20	T	0.0025 s^{-1}	100	20%	20	-	-	-	-	-	-
172	SN	nanofibers	https://doi.org/10.1016/j.jiec.2022.05.085	P(DMAEA-Q-co-NaSS), glass fiber mat	H_2O	M1-0.1	0	6120	?	? x 10 x 1	12	50 mm min^{-1}	700	12.5	-	1400	?	?	50 x 10/20/40	50 mm min^{-1}	JIS-K6251-7	-	-	-	- -	-	-	-	-	-	-	-	-	-
150	DN	helix formation	https://doi.org/10.1016/j.jiec.2022.05.086	P(AAm-*stat*-AAc)–Fe^{3+}/agar	H_2O		75	0.267	?	25 x 4 x 1	?	100 mm min^{-1}	1.55	1410	9.13	1.359	20	Tr	80 x 10 x 1	50 mm min^{-1}	?	0.722	600	T	100 mm min^{-1}	2	21%	600	69%	5 min	96%	20 min	600	25
151	DN	helix formation	https://doi.org/10.1016/j.jiec.2022.05.087	PAAm/agar	H_2O	60 mg/mL A<br>60 mg/mL S	79<br>84	0.642<br>0.447	?	25 x 3.18 x 1	?	100 mm min^{-1}	0.961<br>1.425	1410<br>2340	8.41<br>15.37	3.58<br>3.26	20	Tr	40 x 25 x 1	100 mm min^{-1}	?	3.41<br>2.29	1000	T	100 mm min^{-1}	2	61%	1000	-	-	-	-	-	-
152	DN	helix formation	https://doi.org/10.1016/j.jiec.2022.05.088	gelatin/gellan gum–Na_2SO_4	H_2O	DN-$Sul_{3.2}$-Na_4<br>DN-Sul	49<br>57	42.6<br>2.9	?	?	?	100 mm min^{-1}	7.5<br>2.7	210<br>390	-	27.7<br>9.6	?	PS	?	100 mm min^{-1}	?	-	-	-	- -	-	-	-	-	-	-	-	-	-
154	DN	protein folding	https://doi.org/10.1016/j.jiec.2022.05.089	PAAm,EFK Peptides/4arm-PEG Acrylate	H_2O	PS-DN*	?	-	-	-	-	- -	-	-	-	2.67	?	C	?	? ?	?	-	-	C	16-24 mm min^{-1}	20	93%	50	-	-	-	-	-	-
155	SN	protein folding	https://doi.org/10.1016/j.jiec.2022.05.090	ELPs	PBS	CCP-MCP1	32	0.146	?	40(15) x 10(5) x 2.5	?	2 % s^{-1}	2.6	549	-	1.33	?	Tr	?	? % s^{-1}	?	-	-	T	? % s^{-1}	?	?	?	>95%	0	-	-	150	25
156	SN	protein folding	https://doi.org/10.1016/j.jiec.2022.05.091	PAAm, G8-Protein	H_2O	PAA-G8, 100 mg/mL	37	0.012	?	?	?	10 mm min^{-1}	0.068-0.082	1100	?	0.9	?	PS	?	10 mm min^{-1}	?	?	500	T	5 s^{-1}	5k	?	500	-	-	-	-	-	-
162	SN	host-guest	https://doi.org/10.1016/j.jiec.2022.05.092	PEG Slide Ring Gels	H_2O	SR-0.38	62	?	?	?	?	0.125 s^{-1}	5.5	13400	22	3.6	1	PS	15 x 4 x 1	0.125 s^{-1}	JIS K 6251 No.7	-	-	-	- -	-	-	-	-	-	-	-	-	-
163	DN	host-guest	https://doi.org/10.1016/j.jiec.2022.05.093	poly(β-CD)/Alkyl-bisMA + HEA	H_2O/DMF	EV@CD-1%<br>1,9@CD-1%	>50	?	?	34 x 2 x 1.5	10	1 mm s^{-1}	9.5<br>9.04	1385<br>1600	79.3<br>56.82	66.3<br>60	8.5	Tr	34 x 2 x 1.5	60 mm min^{-1}	?	-	-	T	1 mm s^{-1}	4	?	500	-	-	-	-	-	-
